# Bio-Inspired Architectures Substantially Reduce the Memory Requirements of Neural Network Models

**DOI:** 10.3389/fnins.2021.612359

**Published:** 2021-02-23

**Authors:** Thomas Dalgaty, John P. Miller, Elisa Vianello, Jérôme Casas

**Affiliations:** ^1^CEA-LETI, Université Grenoble Alpes, Grenoble, France; ^2^Department of Microbiology and Immunology, Montana State University, Bozeman, MT, United States; ^3^Insect Biology Research Institute IRBI, UMR CNRS 7261, Université de Tours, Tours, France

**Keywords:** neuromorphic computing, neural networks, backpropagation, computational neuroscience, cercal system, insect neuroscience, escape response, machine learning

## Abstract

We propose a neural network model for the jumping escape response behavior observed in the cricket cercal sensory system. This sensory system processes low-intensity air currents in the animal's immediate environment generated by predators, competitors, and mates. Our model is inspired by decades of physiological and anatomical studies. We compare the performance of our model with a model derived through a universal approximation, or a generic deep learning, approach, and demonstrate that, to achieve the same performance, these models required between one and two orders of magnitude more parameters. Furthermore, since the architecture of the bio-inspired model is defined by a set of logical relations between neurons, we find that the model is open to interpretation and can be understood. This work demonstrates the potential of incorporating bio-inspired architectural motifs, which have evolved in animal nervous systems, into memory efficient neural network models.

## 1. Introduction

Neurons are the fundamental computational units in models of connectionist approaches to artificial intelligence (McCulloch and Pitts, 1943; Hodgkin and Huxley, 1952). They can be understood computationally as defining a linear hyper-plane in an input feature space through the application of an activation function to a weighted sum of these features. While single neurons alone can solve only simple “linearly separable” tasks, networks of such neurons can together solve more complex, non-linear, problems (Minsky and Papert, 1969). The predominant approach in recent years toward developing and training neural networks to solve specific problems has been that of deep learning (LeCun et al., 2015), which can attribute its success to the combination of two important ideas. The first is the universal approximation theorem (Cybenko, 1989) which states that a fully-connected feed-forward hidden layer of neurons is capable of approximating any continuous function—given an appropriate combination of synaptic parameters. The second is the use of batch backpropagation with stochastic gradient descent (Linnainmaa, 1976; Rumelhart et al., 1986) as a means of determining the synaptic parameter values that link together these successive layers of neurons. However, deep learning models with hidden layers are characterized by non-convex loss surfaces. As such, backpropagation converges to a locally-optimal configuration of parameters, not necessarily the global optimum, as a function of their random initialization. In order to improve the quality of these local minimas the solution has been to include many, hence deep, wide neural network layers (Choromanska et al., 2015), although this ultimately leads to unwieldy and memory inefficient models which are difficult, arguably impossible, to interpret (Gilpin et al., 2018).

An important approach within deep learning, first proposed in the 1980's as the “neocognitron” (Fukushima, 1980), has developed into the field of convolutional neural networks (CNNs) which achieve state of the art performance in applications related to image processing. This is in spite of the fact CNNs are composed of a subset of the parameters of a fully-connected feed-forward network of an equivalent depth (Krizhevsky et al., 2012). Crucially, CNNs which were originally inspired by research into the cat visual cortex (Hubel and Wiesel, 1962), and more recently the *Drosophila* visual system (Tschopp et al., 2018), have demonstrated the potential of applying backpropagation to task-appropriate neural network architectures inspired by biology—as an alternative to the use of universal approximators. Other deep learning architectures, namely attention-based models (i.e., Transformers) (Bahdanau et al., 2014) and generative adversarial networks (Goodfellow et al., 2014), have lead to respective leaps in machine translation and novel data-point generation that further reinforce the importance of architectural innovation in neural networks and deep learning.

In the field of neuromorphic computing, where a more “bottom-up” approach is taken to artificial intelligence, research into biological nervous systems has, as in the case of CNNs, also provided a source of architectural inspiration. This has led to models which, for example, incorporate dynamical and topological motifs inspired by the *Drosophila* visual, the honey-bee olfactory, honey-bee central complex, cricket auditory, and cockroach motor systems into models for motion detection (Dalgaty et al., 2018), contrast enhancement (Schmuker et al., 2014), path integration (Stone et al., 2017), temporal pattern detection (Sandin and Nilsson, 2020), and locomotion (Beer et al., 1992), respectively. However, such approaches have been somewhat limited by lack of an effective means of defining model parameters, whereby manual parameter tuning or correlation-based Hebbian learning rules (Hebb, 1949) are typically employed.

In this paper we propose a computational neural network model for the air current evoked jumping escape response studied in the cricket cercal system. Instead of manual tuning or Hebbian learning, we employ the backpropagation algorithm, more commonly used in the deep learning setting, as a means of model parameterization. In contrast with deep learning, however, backpropagation is not used to find an arbitrary local loss minimum based on a random parameter initialization, but instead as a means of steering the parameters toward optimal values consistent with the logical structure of the bio-inspired architecture that relates neurons to one another. We find that, when applied to the detection of a simulated attacking predator, the optimized cercal system model is able to obtain the same performance as multi-layer perceptrons (MLPs), which are based on the universal approximation theorem—although requiring between one and two orders of magnitude fewer parameters. Ultimately, the results provide a strong basis for the incorporation of biologically inspired architectures into memory efficient neural network models and serve as a reminder that intelligence in animal nervous systems is often more than than learning—it also arises from innate, evolved neural architectures that are built-in from birth.

## 2. Materials and Methods

### 2.1. The Cricket Cercal System

**Figure 1:**
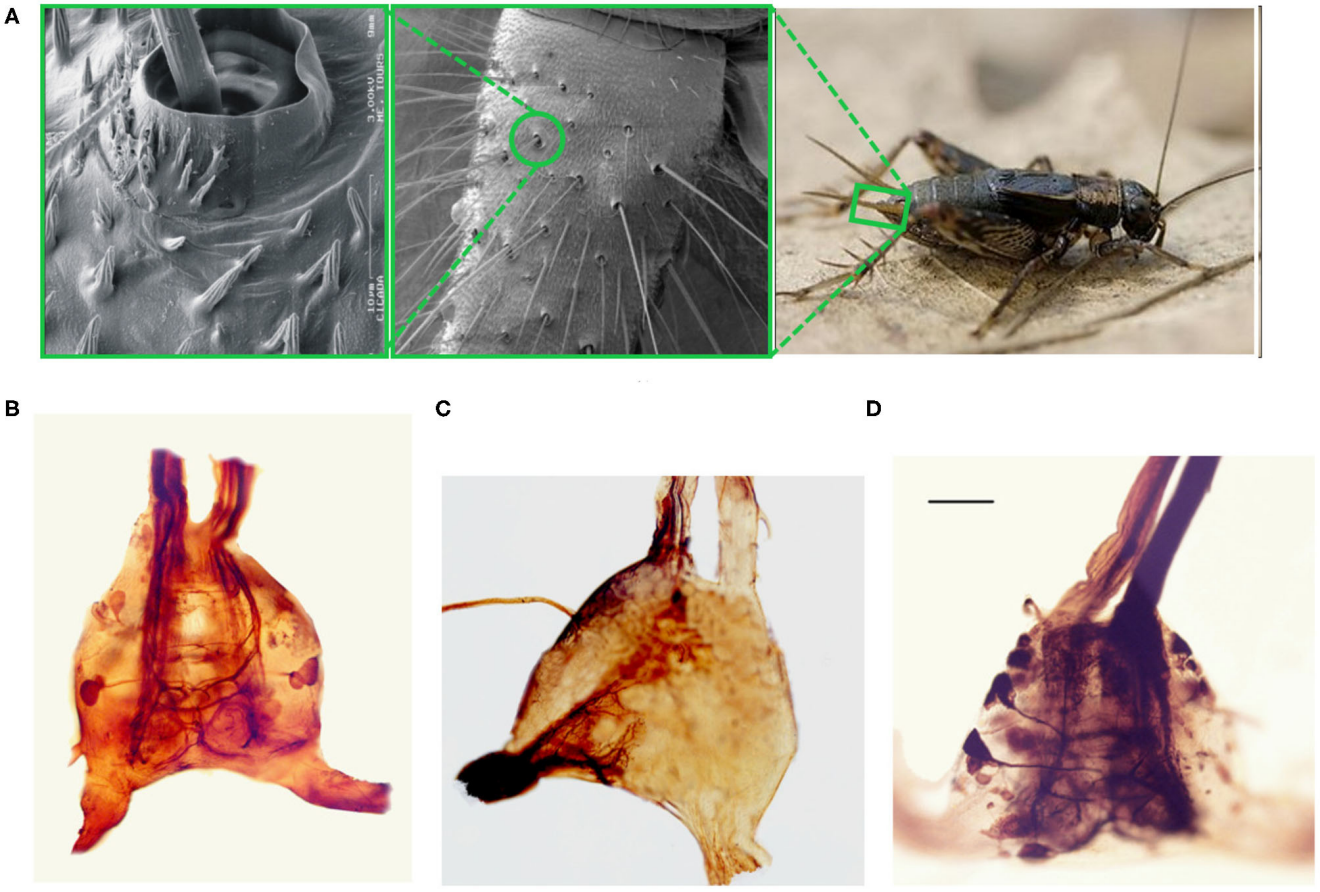
Optical and scanning electron microscopy images of the cricket and its terminal abdominal ganglion. **(A)** Three panel image showing different extents of zoom of the cricket cercal hairs. (Right) An image of a wood cricket. A green rectangle highlights the base of its right cercal appendage. (Center) a scanning electron microscopy image of the base of a crickets cercus. A green circle highlights one of the sockets within which cercal hairs pivot. (Left) A scanning electron microscopy image of a cercal hair embedded within a socket. **(B)** An optical microscopy image of an isolated terminal abdominal ganglion. The two extensions at the base of the image correspond to the cercal nerves, where the sensory neuron axons from the hair receptors on the two cerci project into the ganglion. The two vertical extensions at the top of the image are the two axon bundles composing the nerve chord that ascends up to higher ganglia. **(C)** An image of the TAG where cobalt chloride dye, appearing almost black in high concentrations, has been used to stain the afferents projecting from the left cercus into the ganglion. **(D)** An image of the TAG where dye has been used to stain the cell bodies, dendrites, and the axonal projections up the nerve chord, of a subset of giant interneurons.

The cricket cercal system has been under investigation for several decades, leading to an understanding of many of its neural and biomechanical components (reviewed extensively in Jacobs et al., 2008; Ogawa and Miller, 2019). The nervous system of the cricket is characterized by a chain of ganglia along a nerve cord which runs from the head of the animal down to the rear of the body pictured in **Figure 5B** (Insausti et al., 2008, 2011). Signals from various sensory and motor systems ascend up or descend down the nerve cord between the ganglia where information is processed to mediate the animal's behavior. The interneurons making up the cercal sensory system are contained in the Terminal Abdominal Ganglion (TAG) at the very posterior end of the nerve cord (Figures 1B–D). This cercal sensory system mediates the detection and analysis of air currents which activate sensory receptors on the crickets two rear cercal appendages. These appendages, or cerci, are long antenna-like structures, each of which is covered by up to a thousand filiform hairs as pictured in the electron microscopy image of Figure 1A (Magal et al., 2006; Miller et al., 2011; Heys et al., 2012). Below each hair there is a sensory neuron which, under mechanical displacement of the hair, will fire action potentials that propagate along an axon into the TAG (see Figure 1C). The ensemble of all of the sensory afferents on the two cerci project to specific locations within the TAG and form a sensory feature map (Bacon and Murphey, 1984; Jacobs and Theunissen, 1996, 2000; Paydar et al., 1999). Different spatial regions of this map represent information about air-current direction and velocity transduced around the animals cercal appendages.

Within the TAG, there are approximately twenty large spiking interneurons (Figure 1D), which receive direct excitatory synaptic input from the filiform sensory afferents (Jacobs and Murphey, 1987). These are “projecting” interneurons: they send axons up the nerve cord to higher ganglia (**Figure 5B**). There are also an unknown number of small local interneurons within the TAG which do not send axons up the nerve cord (Bodnar et al., 1991; Baba et al., 1995). Many of these local interneurons are non-spiking, or “graded release,” neurons which interconnect between themselves and the large spiking interneurons. Each of the large spiking interneurons has a unique anatomical projection of its dendritic trees within the sensory afferent map (Jacobs et al., 1986; Jacobs and Theunissen, 1996). As a result, each of these interneurons has a unique specific responsiveness to the different properties of the external air current stimuli. The ensemble activity pattern of these interneurons form a compressed representation of the stimulis which is sent up the nerve cord to higher ganglia. This representation is then, in turn, used to initiate appropriate behaviors in other areas of the nervous system. For this study, we restrict our terminal abdominal ganglion model to neurons and stimuli that concern the escape response behavior.

We have developed a reduced model of the cercal system. It consists of 24 neurons: 16 neurons that represent an integrative input layer, seven neurons in a hidden layer which represents the projecting interneurons within the TAG, and a single neuron in the “output layer,” supposed to exist in a higher motor ganglion in the animal, whose response denotes whether a jumping escape response should be initiated. The characteristics of the neurons in these three layers, and the rationale for their inclusion and inter-connectivity with other neurons, is described in the following sections and diagrammed in **Figure 4**. Before that, however, we introduce the statistical simulation method used to generate the spiking activity of the cricket's ensemble of cercal hairs that act as the input for our model.

### 2.2. Statistical Model of Filiform Hairs

**Figure 2:**
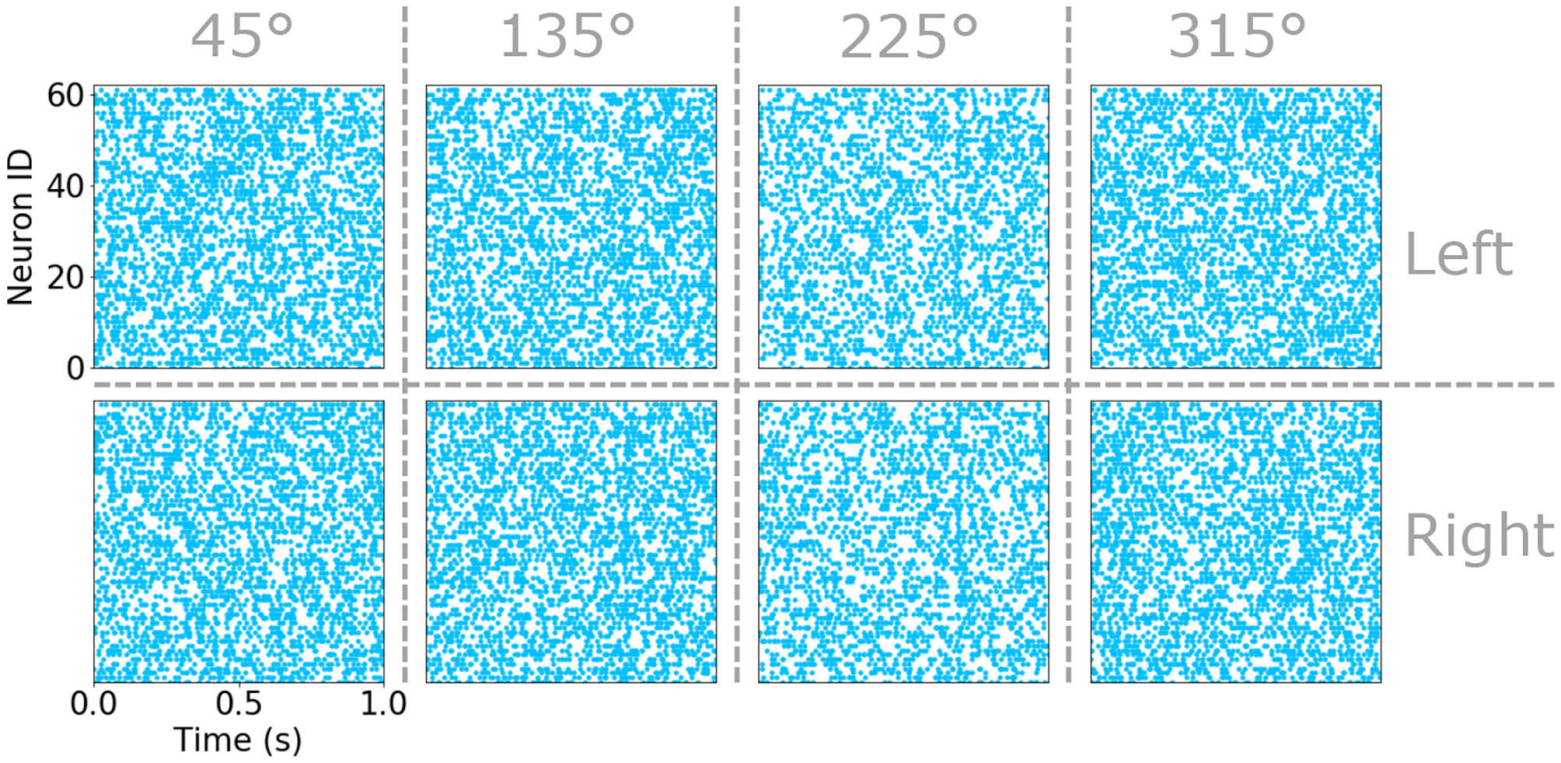
Raster plots of the sensory hair spike time in the eight populations of sensitive to slow air currents. Sub-populations corresponding to the left an right cercus as well as the four preferred directions are indicated by vertical and horizontal dashed lines. Each raster plot marks the spike-time of each neuron in a sub-population (identified as a number between 0 and 59) with a blue point.

In the cricket cercal system, neural processing begins at the cercal filiform hairs. The structure, distribution, biomechanical, and electrophysiological properties of these sensory receptor hairs have been studied extensively in several labs over the previous three decades. Of particular relevance to the modeling studies presented here, the stimulus-response properties of a very large sample of filiform hairs to air current stimuli having different velocities and directions have been recorded with neurophysiological electrodes (Landolfa and Jacobs, 1995; Landolfa and Miller, 1995; Shimozawa et al., 1998; Miller et al., 2011). The data presented in these publications served as the basis for the specification of our model parameters. In particular, Figures 2, 7 of Landolfa and Miller (1995) show the experimental apparatus and an example spike train recorded during experiments that were used as the basis for our simulations.

There are hairs of many different lengths on each cercus, and the length of each hair determines the range of frequencies, or speeds, of air currents to which it is most responsive. Longer hairs are more responsive to slower air currents and short ones to faster air currents. Each filiform hair has a spiking neuron at its base which is mechanically activated when the hair is displaced. These sensory neurons have been observed to fire even under extremely low background air current intensity, at rates of up to hundreds of spikes per second (Landolfa and Miller, 1995). The base of each hair is constrained in a complex mechanical socket such that it can pivot freely along only one specific axis. Characterization of socket orientation distributions on the cerci have revealed four distinct populations—hairs that are sensitive to air currents directions coming from 45, 135, 225, or 315° relative to the head of the animal, which by convention points to 0° . Further studies on the distribution of hair lengths revealed a bi-modal distribution of hairs into two broad but overlapping length categories (Magal et al., 2006). In functional terms, this general distinction can be used to categorize the two groups of hairs as responding more readily to either slow air currents (long hairs) or fast air currents (short hairs). Given that there are two cerci, each containing populations of long and short hairs that are sensitive to four directions of air current, there are 16 sub-populations of cercal filiform hairs which respond optimally to specific, restricted combinations of air current direction and speed. A further study noted another response characteristic of a hair as a function of its length. For an accelerating burst of fast air, shorter filiform hairs respond with a reduced latency with respect to longer ones (Steinmann and Casas, 2017). Whether an anatomical adaptation or a happy coincidence this latency provides a useful signature in the recognition of an attacking predator.

Based on these findings we developed a statistical model for the generation of spikes for sixteen sub-populations of hairs: one for each air current angle (45, 135, 225, or 315°) for each of two hair lengths (long or short) for each of the two cercus (left or right). Each sub-population is composed of *N* hairs.

This model was used to generate individual simulation runs, each one second in duration, of the ensemble spiking activity of all 16 × N sensory neurons. Each simulation is specified by five parameters. Two of these parameters are (1) the *background intensity* and (2) the prevailing *background direction* of the ambient air current. These background air currents correspond to the main source of “noise” that would be expected in a realistic situation that could confound the animal's ability to detect the presence, distance, and direction of a predator. Essentially the background air current imposes a variable level of activity in the receptor cells that, in turn, would lead to a background activity in the interneurons of the TAG. The other three simulation parameters correspond to the simulated predator attack. They specify (3) the *attack angle*, (4) the *attack speed*, and (5) the amplitude of the air current that is assumed proportional to the *attacker size*. For each individual one second simulation, values for these five variables are random-uniformly sampled between a lower and upper bound. An attack stimulus is presented one per simulation, resulting in a peak air-current intensity at either 350 or 700 ms during this one second. The statistical model generates 16 sets of *N* sensory neuron spike-times: one per sub-population.

The specific protocol for defining the responses of individual receptor hairs to an attack stimulus was as follows. For each receptor of each sub-population, the baseline firing pattern due to background air drift within the 1 s interval is set by sampling from a uniform random variable between 0 and 1 s. Each of these samples corresponds to a spike-time. An integer number of such samples are made for each hair according to the *background intensity*: if the intensity of the background air-current is greater, more spike-times are sampled during the simulation to reflect this. The limiting rate for each particular hair was determined by the length and orientation of that hair. Baseline rates of short hairs were half that of long hairs. Furthermore, baseline rates for hairs responding optimally to the direction of the prevailing *background direction* drew additional samples and hairs sensitive to the opposite direction drew fewer. The specific parameters were drawn from earlier published studies (Landolfa and Miller, 1995). At either 350 or 700 ms during the simulation, a simulated attack, originating from an angle, θ, between 120 and 240° (i.e., from behind the animal) occurs. Rather than corresponding to an attack onset, these times reflect instead the point at which the air-current intensity resulting from an attack is at its greatest levels. The attack angle impacts the probability of a hair responding to this attack as a function of its orientation *p*_θ_ and on which cercus (left or right) the hair exists on *p*_*side*_. In order to determine whether a hair responds to an attack, we perform a Bernoulli trial with success probability *p*. If this trial succeeds, an attack is incorporated into the existing set of uniformly distributed firing spike-times by sampling one additional spike-time from a normal distribution aligned with the attack time (either 350 or 700 ms per simulation). Specifically, long hair sensory receptors sample a firing timestamp from a normal random variable centered on either 350 or 700 ms, and the short hair sensory receptors sample from a normal random variable shifted backwards (toward zero) in time by 10 ms—reproducing the response latencies and the effect of a ramping air-current reported in Steinmann and Casas (2017). The standard deviation of the normal random variable is equal to the inverse of the *attack speed*. The probability to sample an additional spike-time from these normal random variables is impacted by an additional parameter corresponding to the attacker size *s*. Ultimately, hair sub-populations with a preferred direction, θ_*p*_, oriented toward the attack angle and the sub-populations on the cercus that are on the same side of the animal as the attack originates from respond more. The probability, *p*, is defined as:

(1) $$p=p_{\theta }\times p_{side}\times s,$$

(2) $$p_{\theta }=(1-\beta_{1})\times cos|\theta_{p}-\theta |+\beta_{1},$$

(3) $$p_{side}=((1-\beta_{2})\times exp(\frac{-|\pi -\theta |}{\alpha })+\beta_{2}.$$

The *attacker size*, *s*, is a number between 0.75 and 1, β_1/2_ are the minimum response probabilities for *p*_θ_ and *p*_*side*_, respectively, and α defines how the response intensity decays on the contra-lateral cerci to the attack as a function of the *attack angle*, θ. An example of the raster plots obtained using this statistical model for the eight sub-populations of sensory hairs under moderate background air-current, a predominant background drift coming from 45° and an attack at 700 ms are plotted in Figure 2. Under closer inspection, faint vertical bars of spike-times align around the time of attack—the result of superimposing the spikes generated from the attack on top of those sampled uniformly during the simulation. These raster plots are consistent with similar observations of the neurophysiology in the real system reported in Landolfa and Miller (1995). Further full examples, resulting from two different sets of input parameters and including the responses of the sub-populations responsive to fast air-currents too, can be found in Supplementary Figures 1, 3.

### 2.3. Input Layer: Feature Map Neurons

In the cricket TAG, this information on air-current direction and speed, represented by the ensemble firing pattern of the sensory neurons, is preserved through sub-population specific projections of sensory neuron spikes to specific locations within a sensory feature map. In each location of this feature map, the thousands of sensory neuron spikes that propagate from the cerci merge into continuous analogue signals which denote the intensity of a particular environmental feature (Bacon and Murphey, 1984; Miller et al., 1991; Jacobs and Theunissen, 1996, 2000; Paydar et al., 1999). TAG interneurons are then observed to read out various properties of this feature map through specific dendritic arborizations in particular spatial locations of the feature map.

We propose to model this feature map using an input layer of 16 neurons. Each of these 16 neurons integrates the spiking input from one of the 16 sub-populations of hairs on the left and right cerci. They also implement a leaky-integration model, in other words a parallel resistor-capacitor circuit fed by a current source. They can be described using the differential equation;

(4) $$\frac{dV_{c}}{dt}=\frac{i_{in}R-V_{c}}{RC}.$$

**Figure 3:**
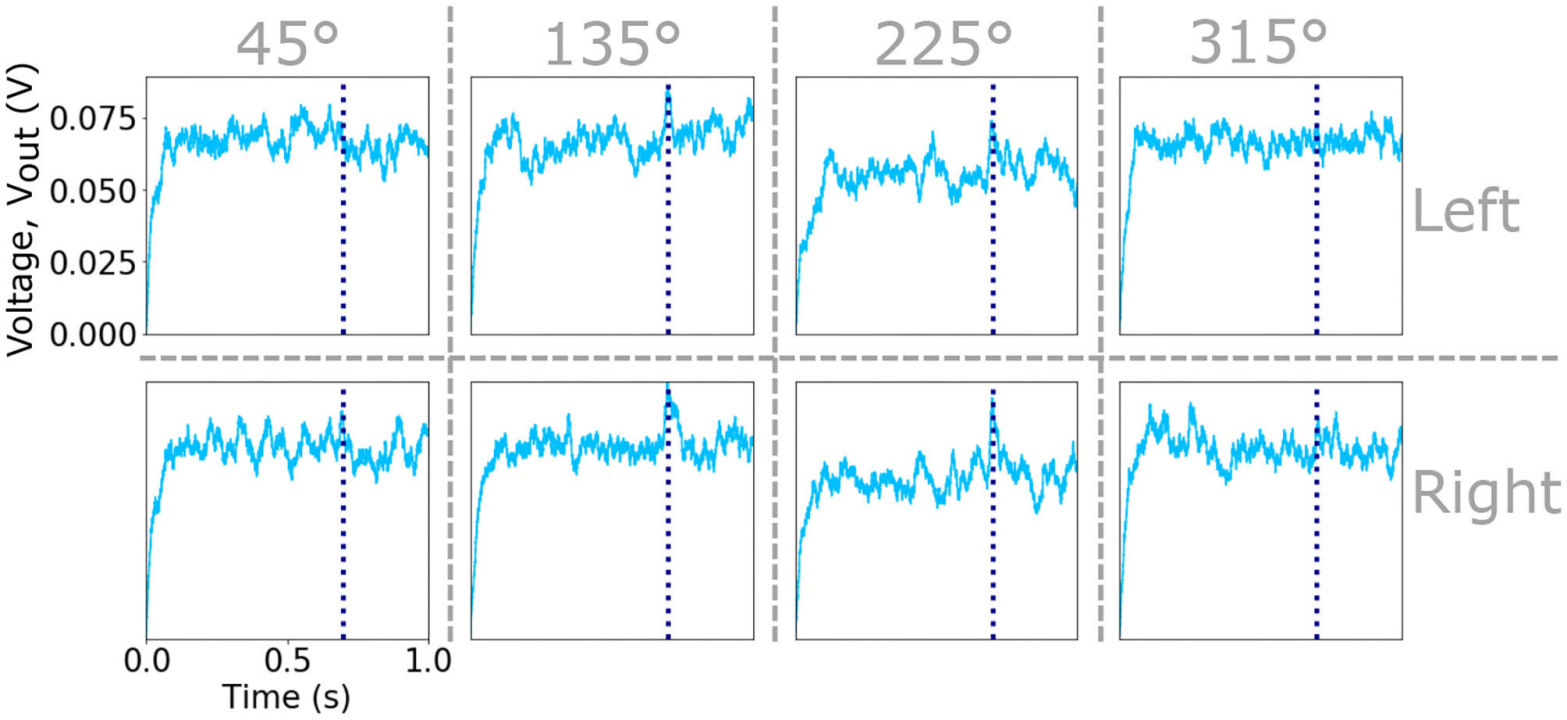
Plots of the *V*_*out*_ signal of the eight input neurons that integrate the spikes from eight sub-populations of sensory hairs. The input neurons receiving excitation from the left an right cercus as well as the four preferred directions are indicated by vertical and horizontal dashed lines. Vertical dashed lines within each plot marks the time of an attack during the one second simulation.

A current *i*_*in*_ is injected into the circuit at each spike-time recorded by the statistical filiform hair model, resulting in a capacitor voltage *V*_*c*_ which decays in time to zero in the absence of an input current. In this paper we use 0.1 pF, 200*GΩ*, and 1 pA as the capacitor, resistor and current pulse values, respectively. This model allows the neuron to integrate temporal information within a time window defined by the product of *R* and *C*. Based on observations of interneurons in the TAG, which appear to receive excitation above and below a set-point due respective increases and decreases from ambient background air-current levels, we apply a hyperbolic tangent function to the difference between the instantaneous capacitor voltage, *V*_*c*_, and its average over the training set, of *D* points, to approximate results published in Landolfa and Miller (1995). In other words, under stimulation by average instantaneous background air currents, the output of the 16 input layer neurons should be close to zero;

(5) $$V_{out}=tanh(V_{c}-\frac{1}{D}\sum_{x=0}^{D}V_{x}),$$

where *D* is the number of training data points. This results in an activation *V*_*out*_ for each of the neurons in the input layer that provides the input for the neurons in the next hidden layer. An example of the evolving activations of the eight input neurons integrating the spike-timing information of the sub-populations of sensory hairs responsive to slow air-currents (due to the raster plots shown in Figure 2) are shown in Figure 3. Vertical dashed lines indicate the time of an attack, in this case at 700 ms. Further examples, showing the activation voltages of all sixteen input layer neurons (due to the raster plots shown in Supplementary Figures 1, 3) are plotted in Supplementary Figures 2, 4, respectively.

### 2.4. Hidden Layer: Cercal Interneurons

**Figure 4:**
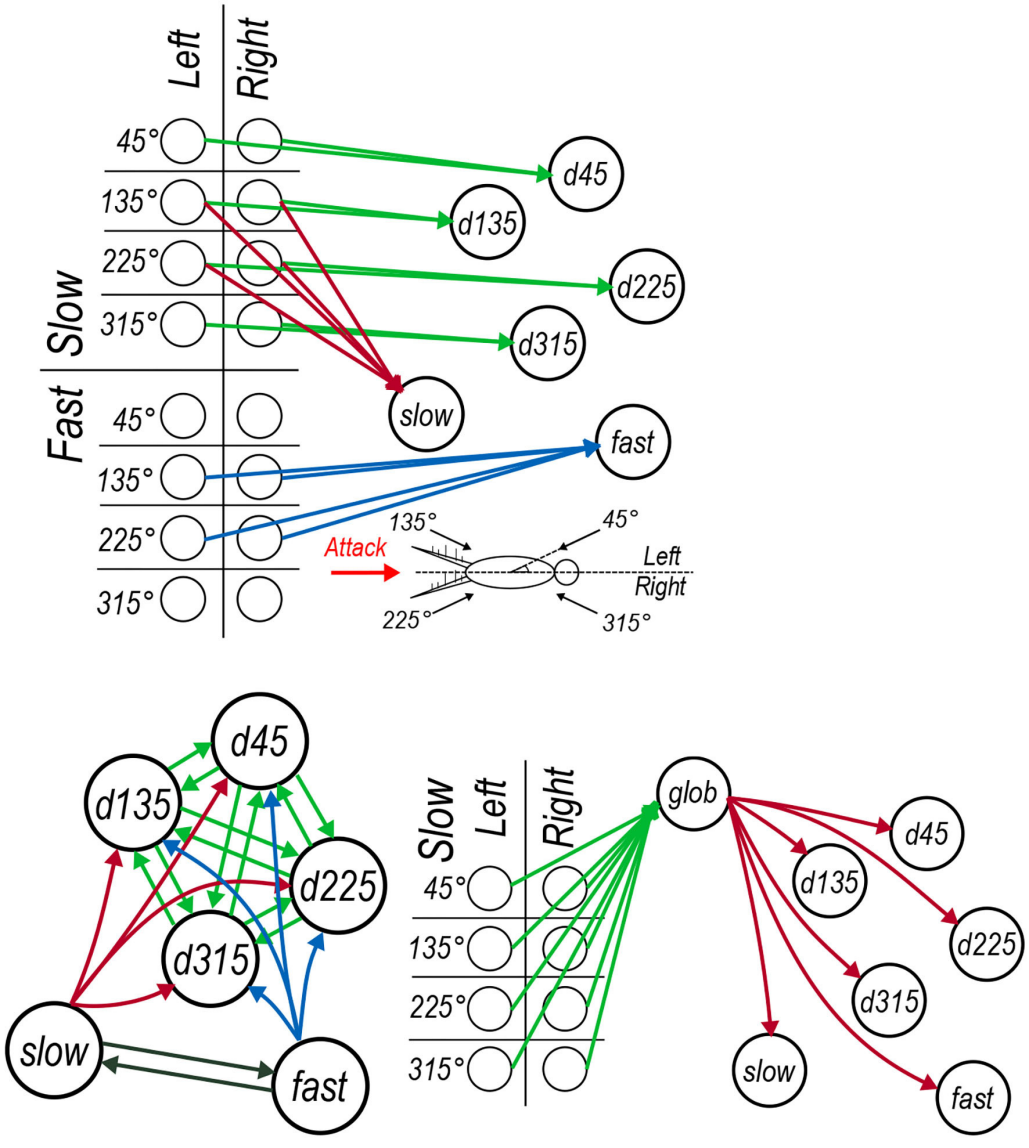
Architecture of the cercal system escape response model. Colors of synaptic connections do not correspond to synaptic inhibition or excitation but instead are used to help distinguish between sets of synapses. **(A)** The feed-forward connections between each of the input layer neurons and the six interneurons each of which encodes a particular property of the input. **(B)** The lateral connections between interneurons, including the (green) mutual connectivity between the directionally selective neurons, (red and blue) the lateral connections from the air speed sensitive neurons onto the directionally selective interneurons and (black) the mutual lateral connections between the air speed sensitive neurons. **(C)** The global regulation network which receives (green) synapses from all of the low frequency air current input layer neurons and sends regulatory synaptic connections to each of the six interneurons.

The hidden layer, borrowing the term from models based on the universal approximation theorem (Cybenko, 1989), is modeled as a seven neuron circuit based on our interpretation of results that have been published in past neurophysiological studies. This group of seven interneurons includes four neurons “tuned” preferentially to four specific air current directions, two interneurons tuned preferentially to different air current speeds (and relatively insensitive to direction of those air currents), and a final interneuron whose activity indicates the overall “global” background air current intensity across all directions. These neurons represent these specific quantities through logical connections to specific subsets neurons in the input layer and, through additional lateral interactions between interneurons, shape an optimal representation that can be used to drive the models outputs. This network architecture is depicted in the three panels of Figure 4. Each of the 16 input layer neurons are identified using a grid which categorizes them by angle, speed, and side in Figure 4. The rationale for the connections between the neuron elements are as follows.

- **Directional interneurons**: Past studies have documented several projecting interneurons that are responsive to low velocity air currents from a very restricted range of directions, and suppressed by air currents from the opposite directions; i.e., they are directionally selective (Jacobs et al., 1986; Miller et al., 1991). These interneurons have been observed to have dendritic arbors in locations of the TAG feature map which receive sensory excitation from air currents from the direction the interneurons is responsive to Bacon and Murphey (1984) and Jacobs and Theunissen (1996). In our model these neurons are labeled as *d45, d135, d225*, and *d315*. Each of these interneurons receive input from the corresponding slow input layer neurons from both the left and right cerci as in Figure 4A.
- **Speed interneurons**: Other interneurons have been observed to be responsive to a much broader range of directions and instead appear predominantly sensitive to different air current speeds from the rear of the animal (Bodnar et al., 1991; Miller et al., 1991). In Figure 4A, these are labeled as *slow* and *fast* that respectively receive input from the input layer neurons encoding air currents coming from behind due to slow and fast stimuli (originating from long and short receptor sub-populations on the cerci, respectively).
- **Global regulation interneuron**: The seventh interneuron in the model is defined as the “global regulation” cell, labeled as *glob* for short in Figure 4C. Extracellular recordings of the ascending spiking activity from the TAG have shown, somewhat counter-intuitively, that the giant interneurons are more active in a relatively calm laboratory setting than in the field, where background air current intensity is considerably greater (Dupuy et al., 2012). That has lead to the proposal of a global regulation, or a background subtraction mechanism, at play in the cercal system. Furthermore, interneurons have been reported in the cricket TAG which are responsive to low speed air currents coming from all directions (Baba et al., 1995). Inspired by these two findings we propose to include the interneuron *glob* in the cercal escape system model as depicted in Figure 4C which receives input from the slow input layer neurons in all directions on the left and right cerci and then sends regulatory synapses to the other six interneurons in the hidden layer.

As well as the specific connection pattern from the input layer neurons to the interneurons in the hidden layer, we also incorporate functional lateral connections between these interneurons. Lateral inhibitory connections have been proposed in the cercal system (Jacobs et al., 1986; Miller et al., 1991) based on observations of direction encoding neurons being inhibited under air current stimuli coming from the opposite direction to which they are sensitive. Detailed neurophysiological data about the neural basis for these interconnections is however very sparse, although several non-spiking local interneurons have been studied (Bodnar et al., 1991; Baba et al., 1995) which could well implement these connections. In our model, we do not explicitly include any extra local interneurons to mediate these lateral interactions. Rather, we define direct all-to-all synaptic connections between each of the four directionally selective neurons and between the two speed encoding neurons in addition to uni-directional synaptic connections from the speed encoding neurons to the four directionally selective ones. These connections are depicted in Figure 4B. Instead of defining these as inhibitory connections we allow their sign and magnitude to be determined through the synaptic parameter optimization process that is described in the following section. Each of these seven interneurons, like those in the input layer, implement hyperbolic tangent activation functions on a weighted summation of their inputs and a bias term. The bias terms of each of the neurons determines the activation of the neuron in the absence of external input, corresponding to the resting membrane potential of the biological cells.

### 2.5. Output Layer: Jump Neuron

**Figure 5:**
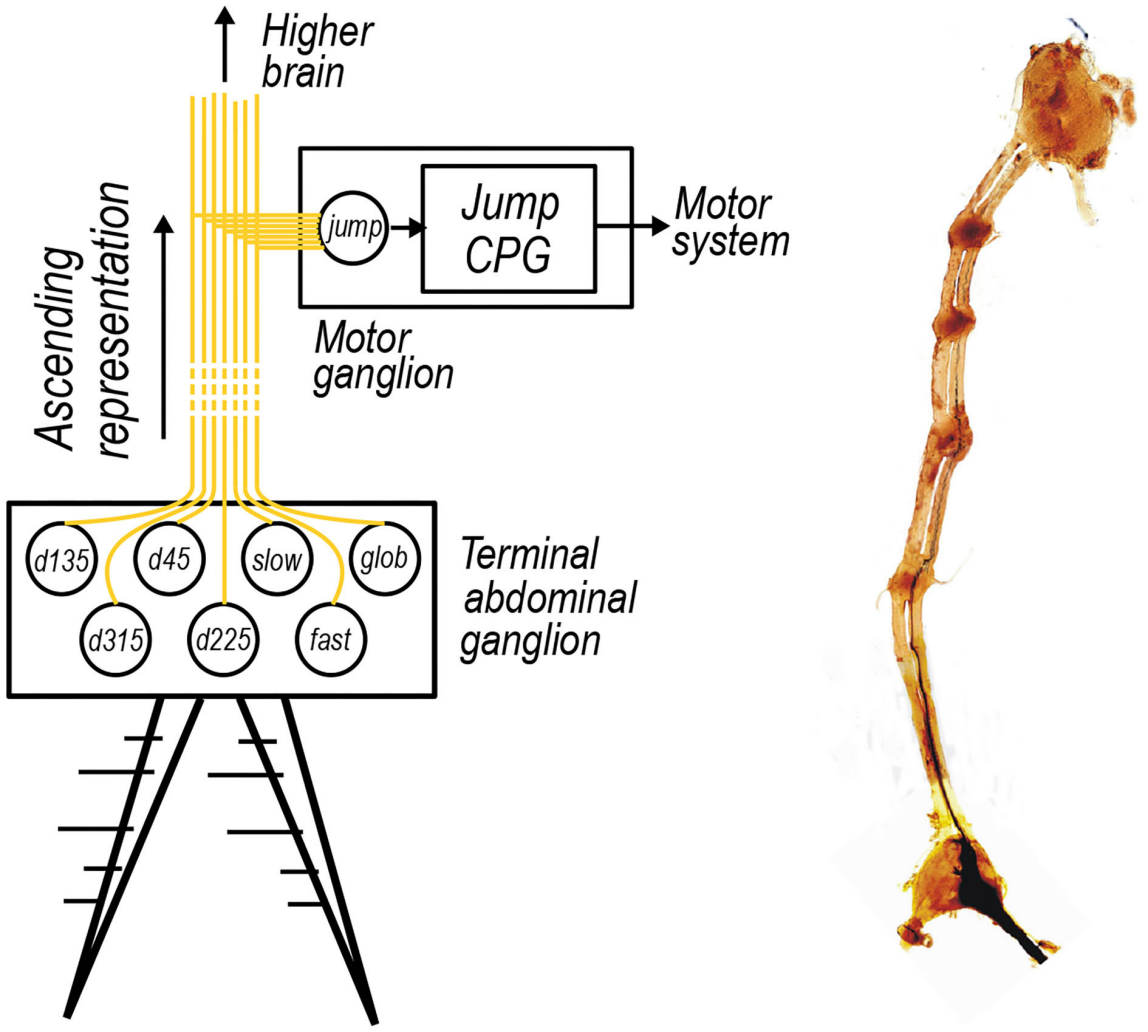
**(A)** The activations of the seven interneurons included in the cercal escape system model ascending up the nerve cord to a higher ganglia. Such a higher ganglion is proposed to contain a neuron or group of neurons, here labeled jump, that looks for certain patterns of activation in the seven interneurons to activate a jump central pattern generator, labeled as jump CPG, to initiate an escape motor routine. **(B)** An optical microscopy image of the ganglion chain of the wood cricket, stained using a cobalt chloride dye. The terminal abdominal ganglion sits at the base of this chain which ascends up to the central brain contained within the head of the animal. Intermediate ganglion, which for example control one of the insects motor systems, appear as more densely stained red dots along the chain.

In the animal, it is supposed that the resulting cercal representation ascends to a higher motor ganglion, one of those pictured in Figure 5B, which, given a certain activation pattern, triggers a central pattern generator to coordinate an escape motor response. Therefore, we propose that each of these seven interneurons in the hidden layer of the model synapse onto a final *jump* neuron as depicited in Figure 5A.

In our model, the jump neuron implements a sigmoid activation function on the weighted sum of the interneuron activations, and a bias term. In this fashion, the output can be viewed as the probability that the original ensemble spiking activity pattern of the sensory neurons on the two cerci represents the signature of an attacking predator.

### 2.6. Model Training

In order to determine the synaptic parameters that connect the input layer neurons to the interneurons, the interneurons to the *jump* neuron as well as the lateral connections between the interneurons, we apply the backpropagation algorithm (Rumelhart et al., 1986) in the context of a supervised machine learning problem. An input dataset was generated by running 1,000 independent simulations of the described statistical model of the cricket filiform hairs. In order to be compatible with our first order model, that does not describe temporal dependencies, the resulting waveforms that are generated by the neurons in the input layer are sampled after the potential attack events at 350 and 700 ms—providing two static snapshots per 1 s simulation. As a function of whether the attack occurs at 350 or 700 ms, these two vectors of 16 instantaneous input neuron activations, are labeled as 1 (attack) or 0 (ambient). Each of these points is also labeled with a prevailing wind direction corresponding to 45°, 135°, 225°, or 315°. This results in a labeled dataset of 2,000 points, each described with 16 features. The dataset was randomly shuffled and then cut into training and testing sets of equal size.

A multi-objective cost function was defined as the sum of the binary cross-entropy loss of the jump neuron, with respect to the attack or ambient label, and the categorical cross-entropy loss of the four directional sensitive interneurons. The losses were combined with equal weight. Intuitively this optimizes the model to simultaneously detect an attack signature and determine the prevailing direction of the background air currents. An additional weight decay (*L*_2_-norm regularization) term (Krogh and Hertz, 1992) was summed with these two losses in order to discourage the optimization from converging to a solution with large parameters values and, possibly, over-fitting to the test data split. The loss function is therefore written as:

(6) $$\begin{array}{l} L=-\frac{1}{N}(\sum_{N}y_{jump}^{*}\ln y_{jump}+(1-y_{jump}^{*})\ln(1-y_{jump})\\ \;+\sum_{N}y_{dir}^{*}\cdot \ln y_{dir}+\sum_{W}\frac{\lambda }{2}||w|{|}^{2}), \end{array}$$

where $y_{jump}^{*}$ and *y*_*jump*_ and $y_{dir}^{*}$ and *y*_*dir*_ refer to the respective labels and neuron activations of the jump output and direction encoding vector, *N* to the training data points of the mini-batch, *W* to the number of weights, *w*, of the neural network and where ln is the natural log operator.

In order to implement the lateral connections between the interneurons without introducing cyclic loops into the model, these synaptic connections were “unrolled by one step.” This is required to avoid the backpropagated gradients from circling indefinitely. This was achieved by applying the activation function to the weighted sum of the inputs from the input layer neurons and the *glob* neuron in a first step and then applying, in a second step, the same activation function to the sum of the first activation with the weighted sum of the lateral connections and the bias term.

The derivative of the mean of this loss over a mini-batch of eight training data points was used to update model parameters inline with the root mean square propagation (RMSprop) stochastic optimization algorithm (Tieleman and Hinton, 2012) over fifty training epochs. The PyTorch automatic differentiation python framework was used to build and train the proposed TAG model (Paszke et al., 2017). The TAG model parameters were not initialized randomly at the beginning of the training process. Rather, initial values of one, zero and negative one are deterministic assigned to each parameter are set inline with the logical structure of the model—for example the synapses connecting *glob* to the other interneurons were initialized to negative one. This was determined to be important in allowing the model to converge to an optimal configuration in 50 epochs as in the random seeding study of Supplementary Figure 5.

### 2.7. Multi-Layer Perceptron Models

In order to compare the test accuracy and memory requirements of the optimized TAG model, single and three fully-connected hidden-layer neural network models, characteristic of a generic deep learning (LeCun et al., 2015) approach, are used. These were also implemented using the PyTorch framework. They were trained using the adaptive moment estimation optimization (Adam) algorithm (Kingma and Ba, 2014). The models were trained over one thousand epochs using the same mini-batches as the TAG model. On average this was observed to be the number of epochs required for the parameters of the larger among the MLPs to converge to a loss minimum (see Supplementary Figure 6). It is also informative to note that this represents an order of magnitude more epochs that required to train the TAG model—requiring only fifty epochs (see Supplementary Figure 5).

In order to prune the MLPs, the distribution of the absolute values of the weights was sorted into an ascending order and the weight values below a certain percentile were set equal to zero. To train the sparsified models, the sum of the *L*_1_ norm of the weights (Lasso regression) was added to the loss function of the model (Tibshirani, 1996). The derivative of this term has the effect of forcing a considerable number of weights close to zero—resulting in a sparse MLP.

## 3. Results

### 3.1. Model Evaluation

In order to evaluate the proposed terminal abdominal ganglion (TAG) cercal escape system model we use receiver operating characteristic (ROC) curves. A ROC curve plots the true (TPR) and false positive rate (FPR) as the probability threshold, that rectifies the activation of the sigmoidal *jump* neuron into a jump (1) or don't jump (0) output, is increased from zero to one in regular steps. To reflect the imbalance between the consequences of not detecting true positive (succumbing to a predator) and responding to false positives (wasting a relatively unimportant amount of energy; Bennet-Clark, 1975). We define the metric “tolerated” FPR, which is the false positive rate that must be tolerated in order to achieve a minimum true positive rate, to assess the model. We fix the minimum TPR to 0.95 in this evaluation.

**Figure 6:**
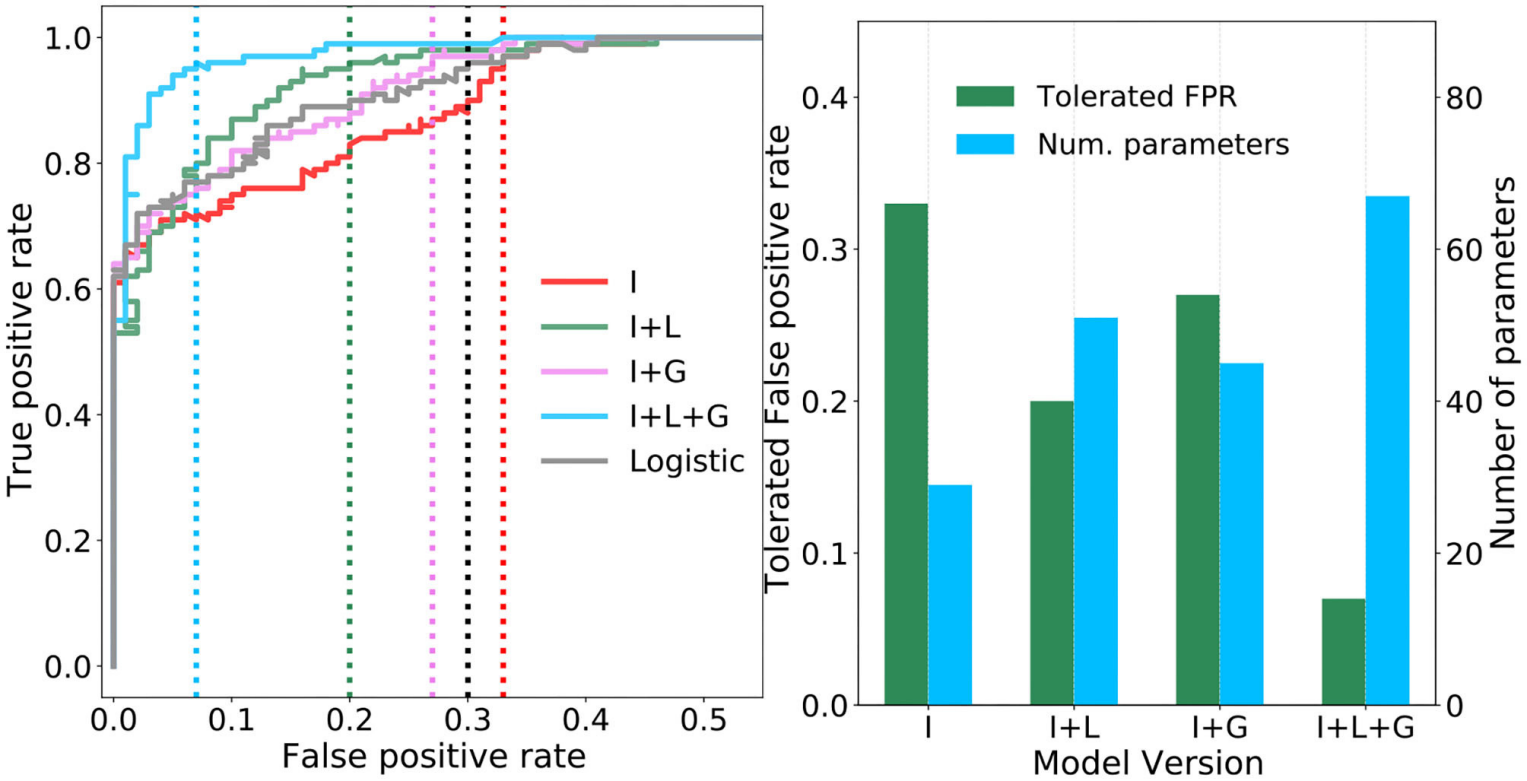
Performance of the four ablated versions of the TAG model. **(A)** Four receiver operating characteristic (ROC) curves are plotted showing how the true and false positive rates vary as the probability threshold on the sigmoidal output *jump* neuron is increased. Vertical dashed lines show the tolerated false positive rate (FPR) for a minimum true positive rate of 0.95. **(B)** Pairs of bar plots for each ablated version of the TAG model show the tolerated FPR given a minimum TPR of 0.95 (green bars, left y-axis) and the number of parameters required by the model version (blue bars, right y-axis).

Figure 6A plots the ROC curves resulting from an “ablation” study of the proposed TAG model, whereby combinations of individual components are considered separately in four different versions (I, I+L, I+G, and I+L+G) of the model. Specifically, the curves correspond to I—only the interneurons, I+L—the interneurons plus their lateral connections, I+G—the interneurons plus the global regulation network, and I+L+G—the interneurons plus the lateral connections as well as the global regulation network. For means of comparison, the ROC curve of a logistic regression model, effectively a single neuron taking input from all sixteen feature map neurons, is plotted in black. In the case of model I, it is observed that, despite increasing the number of neurons from one (i.e., the logistic regression model) to seven, there is a decrease in the tolerated FPR. This indicates that the extraction and combination of specific features alone does give rise to an effective model. In model I+L, the addition of lateral connections between the interneurons is seen to greatly reduce the tolerated FPR and noticeably increase the area bounded under the ROC curve. This demonstrates the importance of allowing neurons, each encoding different properties of the total sensory landscape, to communicate and compete via lateral synapses—increasing the contrast between their responses. In model I+G, the lateral connections are removed and the global regulation network is added. While the tolerated FPR deteriorates with respect to model I+L, there is still a slight improvement with respect to the ROC curve of the logistic model and greatly improved with respect to the neurons acting alone. This suggests a further importance in regulating the activations of the interneurons as a function of the background air current intensity which, intuitively, should make it easier for the *jump* neuron to recognize the activation pattern of an attack superimposed on top of fluctuating background air currents. Finally, in model I+L+G, the full network model is optimized: all lateral connections and the global regulation mechanism are included. The resulting ROC curve, plotted in blue, bounds noticeably more area than any of the other models and achieves tolerated FPR of 0.07—well below that of the other models. This demonstrates that, while the lateral and global connections independently provided modest gains with respect to the logistic model, it is their interplay and the simultaneous application of contrast enhancement and background subtraction that gives rise to the most adept model. While lateral connections are a commonly observed feature in animal nervous systems, this result provides a foundation for the proposal in Dupuy et al. (2012) that a global regulatory mechanism is present in the cricket cercal system. The tolerated FPR of each model version, as well as the number of synaptic parameters required per model, are summarized in the bar-chart of Figure 6B. Here it is noteworthy that, for a relatively modest two-fold increase in the number of parameters, the tolerated FPR reduces by more than three times.

### 3.2. Comparison With Multi-Layer Perceptrons

**Figure 7:**
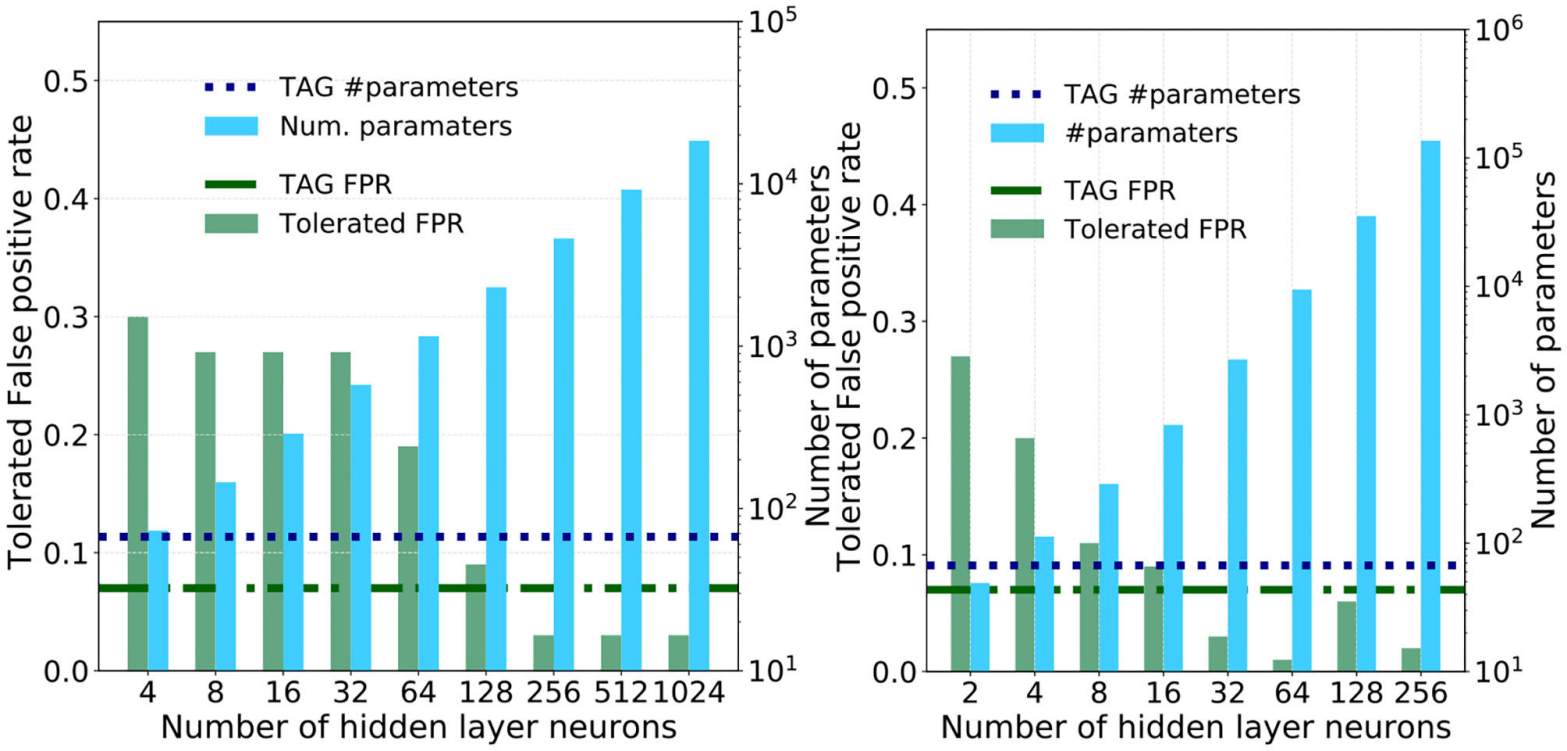
The performance of the single and three hidden-layer multi-layer perceptrons used as a means of comparison against the TAG model. **(A)** Pairs of bar plots over a range of hidden layer sizes for a single MLP. The bars show the tolerated FPR (green bars, left y-axis) and the number of parameters required by the model version (blue bars, right y-axis). Dashed horizontal lines show (green) the TAG model tolerated FPR and (blue) the number of parameters required in the TAG model. The minimum TPR is 0.95. **(B)** Pairs of bar plots over a range of hidden layer sizes for a three hidden layer MLP. The bars show the tolerated FPR (green bars, left y-axis) and the number of parameters required by the model version (blue bars, right y-axis). Dashed horizontal lines show (green) the TAG model tolerated FPR and (blue) the number of parameters required in the TAG model. The minimum TPR is 0.95.

To understand the utility of this bio-inspired TAG model architecture it will be informative to compare it against the tolerated FPR and memory requirements of a multi-layer perceptron derived through the universal approximation approach; whereby the synaptic weights that link successive layers of neurons in a fully-connected feed-forward fashion are optimized using backpropagation (Rumelhart et al., 1986; LeCun et al., 2015). The number of neurons per hidden layer, where each hidden layer is composed of the same number of neurons, is increased logarithmically and trained and tested using the same train and test data sets as with the TAG models. The resulting tolerated FPR, as well as the number of parameters, for each of these different sized models is plotted in the bar-plots of Figures 7A,B for the single and three hidden layer models, respectively.

The tolerated FPR and the number of parameters in the TAG model I+L+G are plotted as horizontal green and blue dashed lines for means of comparison. What is striking is that, for the multi-layer perceptrons to obtain an equivalent performance to the TAG model I+L+G, they are seen to require between one and two orders of magnitude more synaptic parameters. Specifically, to match the TAG model performance, the single hidden layer neural network is seen to require between 128 and 256 neurons and, for the three hidden layer model, between 16 and 32 neurons per layer.

**Figure 8:**
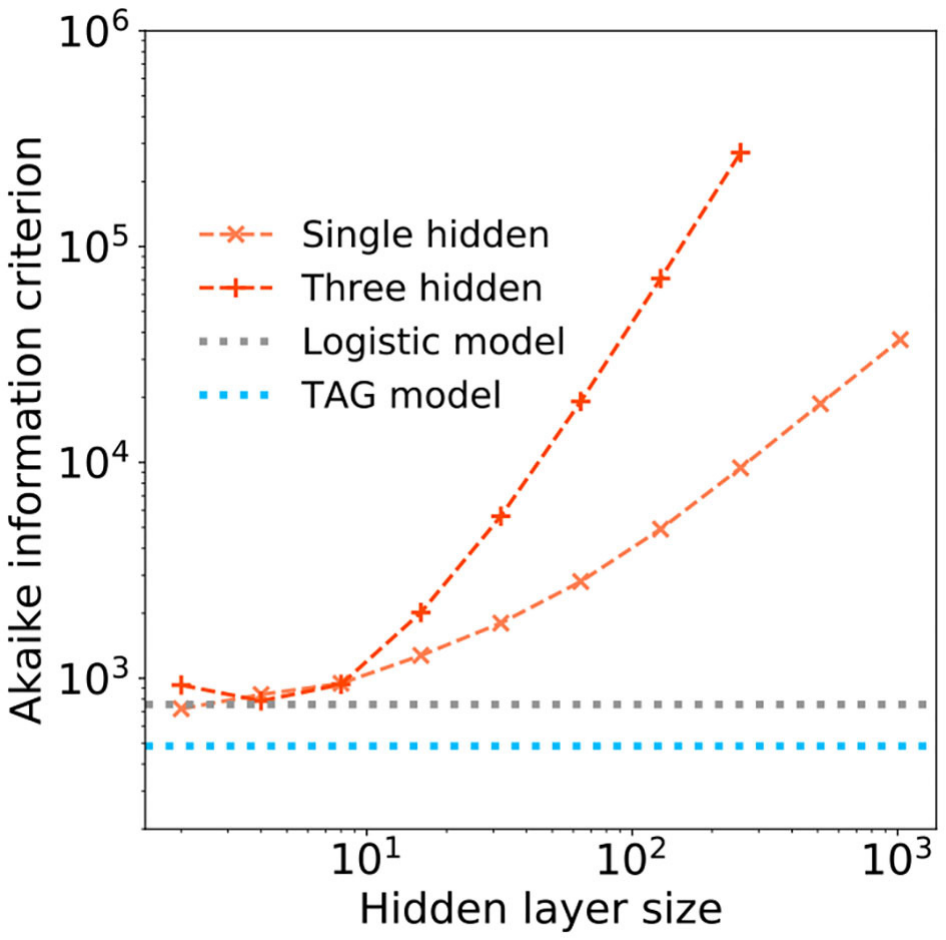
The Akaike information criterion (AIC) is plotted in log-log scale over range of hidden layer width for (orange) a single hidden layer and (peach) a three hidden layer deep learning models. Horizontal dashed lines show the AICs of a (gray) logistic regression model and (blue) the TAG model.

However, as the number of hidden layer neurons are further increased, the tolerated FPR of these larger, more memory intensive, models drops below that of the TAG model—in some cases obtaining a tolerated FPR of 0.03. To understand whether this investment in memory is worthwhile, we use the Akaike information criterion (AIC) (Akaike, 1998). The AIC provides a number proportional to the sum of the model size and the negative log-likelihood of the model and as such offers a means of comparing the efficiency of each solution—a lower AIC indicates a more efficient model. The AIC of the single and three hidden layer MLPs are plotted as a function of the hidden layer size in Figure 8. Additional horizontal dashed lines show the AIC score of the logistic regression (gray) and TAG models (blues). It can be clearly seen that the TAG model is comfortably the most efficient solution to the problem and that, for the extremely large deep learning models which obtain the lowest tolerated FPRs, the AIC score explodes owing to the huge complexity of the model. Furthermore, it is indicative to note that, even for small hidden layer sizes, it is difficult to justify the choice of a model based on the universal approximation theorem model over the use of a lower complexity logistic regression model.

**Figure 9:**
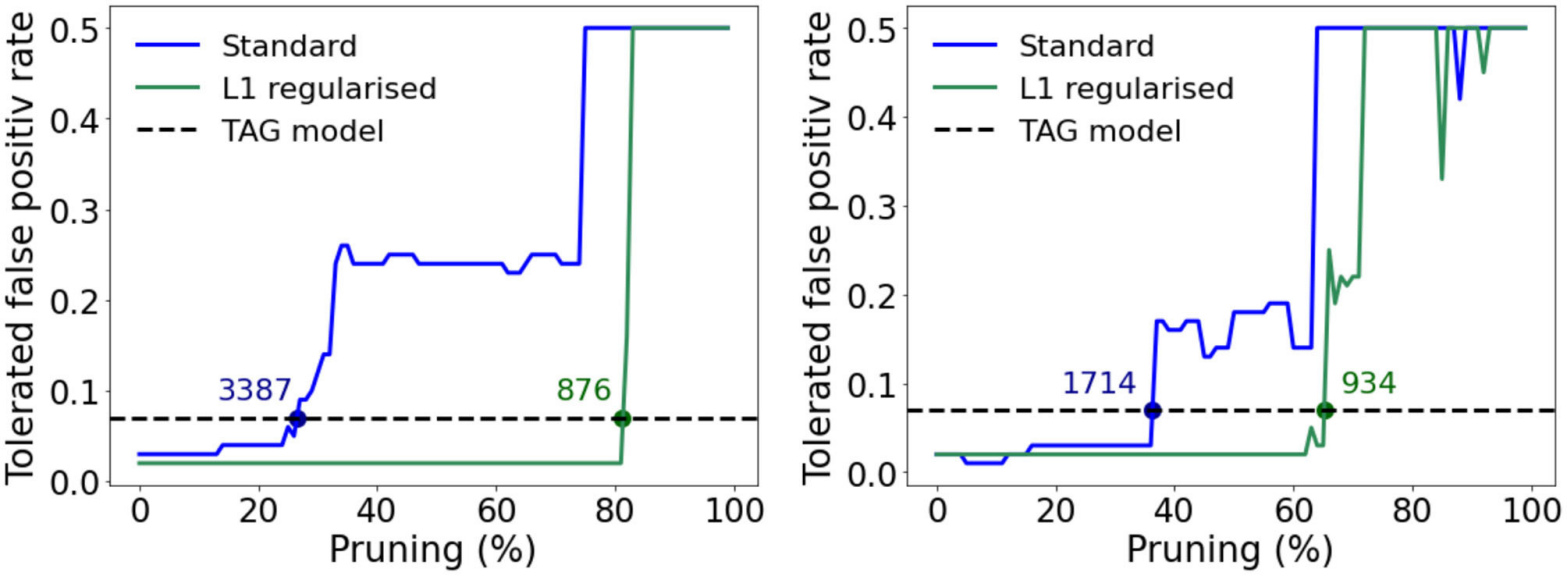
Relationship between the tolerated false positive rate and the percentage of the weights closest to zero pruned for the standard (blue) and *L*_1_-norm regularized (green) models. The horizontal dashed line shows the tolerated false positive rate achieved by the TAG model. The number associated to each dot, drawn when the relationship crosses the horizontal dashed line, denote how many parameters the model required to match the tolerated FRP for the TAG model. **(A)** For the case of a single hidden layer MLP with 256 neurons in the hidden layer. **(B)** For the case of a three hidden layer MLP with 32 neurons per hidden layer.

In order to complete this comparison, we extend it to the case of pruned multi-layer perceptrons—whereby memory requirements can be reduced by deleting synaptic weights with optimized values close to zero (LeCun et al., 1989). Specifically we consider a pruned version of the MLPs in Figures 7A,B and an additional version of each trained where an *L*1-norm regularization term is summed with the loss function in order to enforce sparsity in the resulting model (Tibshirani, 1996). The weight distributions of these MLP models are plotted in Supplementary Figure 7 where it can be seen that the models trained using the *L*_1_-norm loss function have weights grouped very tightly around zero. The tolerated FPR as a function of the percentage of pruned weights in the single and three hidden layer MLPs containing 256 and 32 neurons in each of their hidden layers, respectively is plotted in Figures 9A,B. These dimensions were selected in each case since this was the number of neurons per hidden layer, per MLP, that allowed a lower tolerated false positive rate than the TAG model. In each case, as the extent of pruning increases, the tolerated FPR, after a varying period of robustness, also increases—the point at which the tolerated FPR exceeds that of the TAG model is indicated using a labeled dot in Figures 9A,B. While the *L*_1_-norm regularized models permit a considerable reduction in the number of weights with respect to the standard MLP before a degradation in the tolerated FPR is observed, the number of parameters required in order to maintain a performance equivalent to that of the TAG model architecture was still over an order of magnitude greater than our bio-inspired architecture.

### 3.3. Model Interpretation

In addition to their low memory efficiency, models like MLPs which are based on the universal approximation theorem suffer from another major drawback in their lack of interpretability (Gilpin et al., 2018). Namely, it is difficult to ascertain as to why certain combinations of input features lead to certain output predictions as a function of the cascading layers of synaptic weights determined via backpropagation. This poses ethical and practical problems in many applications of artificial intelligence such as that addressed here which, certainly from the perspective of the cricket, can be considered as a safety-critical application whereby taking action based on the model outputs entails potentially dangerous consequences (i.e., not jumping in the presence of an attacking predator). In contrast to the wide fully-connected layers of neurons in MLPs, the proposed TAG model contains eight neurons and 63 synapses—each defining a set of logical relationships between the neurons which each have well-defined functional roles. As such, based on the optimized synaptic weight values, we make a first-order structural interpretation of the TAG model.

**Figure 10:**
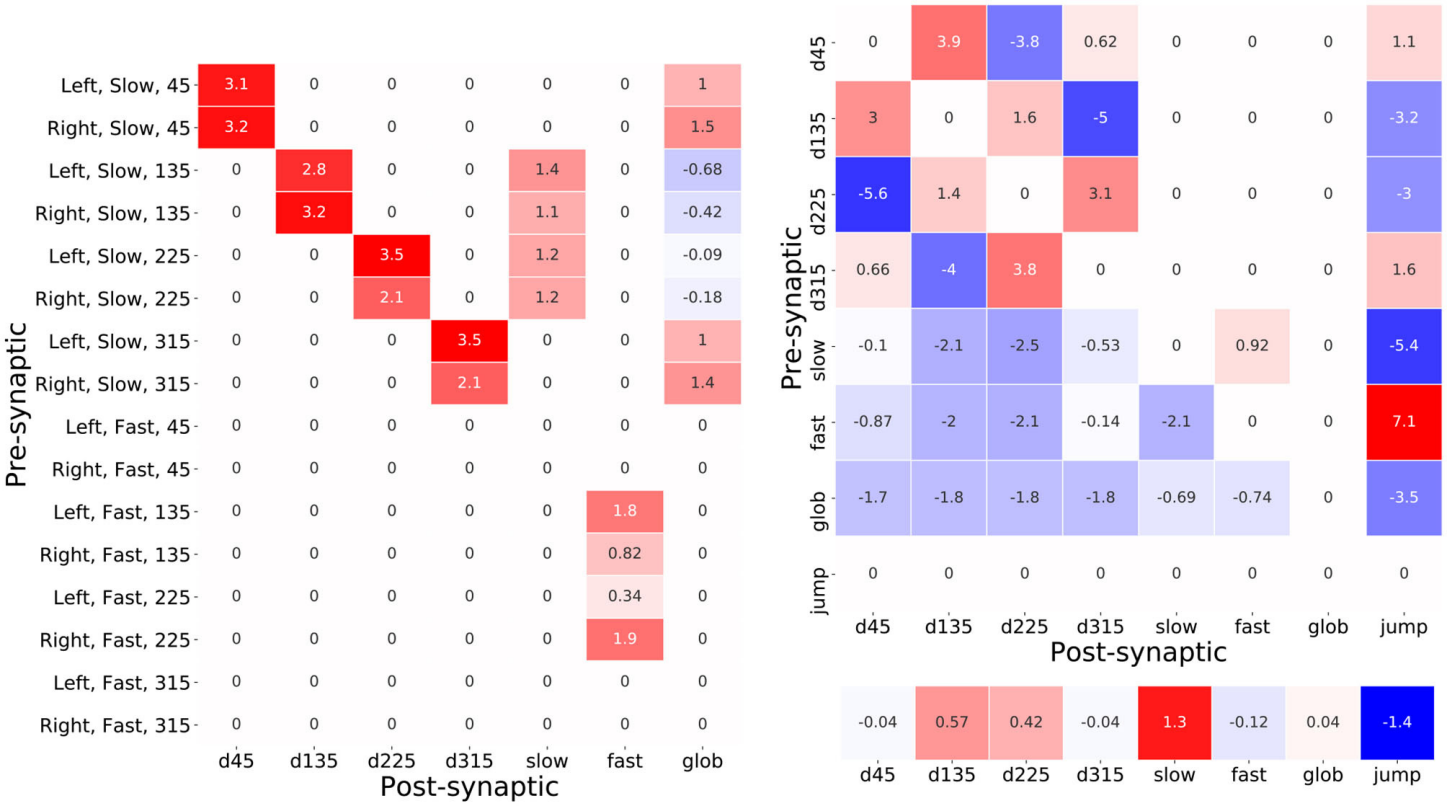
Tables of synaptic weight parameters between pre- and post-synaptic pairs of neurons. The number in each cell shows the value of the weight and the color of the cell denotes the sign and magnitude of the weight. Red cells indicate positive weights and blue ones negative weights. **(A)** The table of synaptic weights between each of the sixteen input layer neurons and the seven interneurons. **(B)** The table of synaptic weights used for each pre- and post-synaptic interneuronal connection. The values of the interneuron biases, corresponding to their resting values, are shown below the table.

A table of these optimized synaptic weights which interconnect the input layer neurons with the interneurons is shown in Figure 10A. Each cell in the table contains the synaptic weight value resulting from the backpropagation based training. The majority of these feed-forward connections are excitatory such that, without consideration of the lateral connections, the interneuron activations would be proportional to the activations of the input layer neurons that connect to them. Therefore, the activation of each of the directional neurons for example, encodes the instantaneous intensity of slow air currents coming from each of the four input air current directions. It is interesting to note the imbalance in the excitation received from the left and right cerci in the cases of *d135, d225*, and *fast* whereby sensory activity coming from 135° is stronger from the left cercus and activity from 225° is stronger from the right. This in fact appears to model the dependency on attack direction described in Equations (2) and (3) whereby attacks coming from the left-hand side excite the sensory neurons oriented at from 135° more; and those from the right-hand side excite the 225° sensory neurons more.

Elevated co-activations of *d135, d225*, and *fast* will therefore correlate with an attack event—in particular the activation of *fast*. Another feature that stands out is the weak negative feed-forward connections from the input neurons, corresponding to air-flow from attack angles, that help decouple the high air-currents resulting from an attack and the activation of *glob* which is intended encode the overall background air-current intensity. Intriguingly, the inhibition coming from the 135° input neurons on the left and right cerci is, in this case, elevated with respect to that coming from 225°. This angular asymmetry is also observed in the weak excitatory inputs from 135° and 225° inputs from the right and left cerci, respectively that are pre-synaptic to the *fast* neuron and, for the authors, it unclear what is the purpose of this subtle adaptation.

A second pair of tables in Figure 10B shows the sign and weight of the optimized synaptic weights inter-connecting the hidden layer neurons as well as their optimized biases. An immediately eye-catching feature is the symmetry observed in the connectivity pattern between the direction encoding interneurons. These interneurons are all seen to strongly inhibit their opposing counterpart—for example the complimentary negative synaptic connections between *d45* and *d225*. Excitingly this is consistent with predictions that have been made regarding the biological system whereby the reduction in directional interneuron activity, when presented with an air-current stimulus contrary to its preferred direction, has been attributed to the existence of laterally inhibiting interneurons between them (Miller et al., 1991). Furthermore, these hidden layer interneurons send excitatory lateral connections to their ipsa-lateral and contra-lateral direction encoding neurons—notably the excitation toward the contra-lateral neuron is consistently greater. Based on the presence of the predicted lateral inhibitory interactions, this result hints at the possible existence of additional lateral excitatory connections in the biological system.

The post-synaptic connection weights of *glob* onto the six interneurons seen in Figure 10B suggest a very interesting functional role of this neuron. Since these weights are negative and *glob* implements a hyperbolic tangent activation function, therein activating negatively for low *background speed* and positively for high *background speed, glob* acts as an excitor given lower background air current levels but, intriguingly, as an inhibitor when the background levels are higher. This latter effect results from the positive product of the negative neuronal activation and the negative synaptic weight value. This is once again an exciting result given the prediction on the presence of a global regulatory or background subtraction mechanism in the biological system (Dupuy et al., 2012).

Finally, it is informative to read off the synaptic weights connecting the hidden layer interneurons to the output *jump* neuron as a logical “bar-code”—offering a means of understanding what combinations of interneuron activations lead to certain output responses. The *jump* neuron is inhibited due to pre-synaptic connections from *d135, d225*, and *glob*. This is likely an adaptation that allows the model to reduce the false positive rate under high background air current levels or high prevailing air-currents originating from the same directions as an attack. Another noteworthy feature of this synaptic bar-code is the opposing strong inhibition and strong excitation from the pre-synaptic *slow* and *fast* interneurons. Once again, since the neurons implement hyperbolic tangent functions, the respective negative and positive co-activation of *slow* and *fast* will result in a joint strong positive excitation of *jump*. This suggests that a key distinguishing feature of an attack is the divergence between the activations of *fast* and *slow*. The optimized TAG model further enhances this divergence in fact using the lateral connections between *slow* and *fast*. In the hidden layer, *slow* synapses positively onto *fast*, and *fast* negatively onto *slow*—the more positive the activation of *fast* therefore, the more negative the resulting activation of *slow* and vice versa. For a similar functional interpretation of the TAG model (see Supplementary Note 1).

## 4. Discussion

The objective of this work was to develop a neural network model of the jumping escape response of the cricket cercal system. Although the model architecture was inspired from research into the biological system, several trade-offs were made from the standpoint of practicality. For example, the sensory feature map, realized in the animal through a complex network of sensory afferents, was simplified into sixteen leaky-integrating hyperbolic tangent neurons. Despite deviating from the exact biology, these practical modeling choices were still able, however, to capture the fundamental underlying computing principles of the system. This highlights the difference between bio-mimicry and bio-inspiration, at least in the context of neural network modeling: the former aims to precisely reproduce biology while the latter takes inspiration from its key principles without necessarily reproducing them in the same fashion. It is interesting to note that these design choices also result in a technologically-plausible model that naturally lends itself to a future silicon neuromorphic implementation. The required hyperbolic tangent, sigmoid and leaky-integration functions are readily implemented using numerous analogue or digital silicon circuits (Lansner and Lehmann, 1993; Indiveri et al., 2011; Davies et al., 2018; Dalgaty et al., 2019). Similarly, the event-based input generated by the models sensory neurons can be realized through the use of delta-modulator circuits (Corradi and Indiveri, 2015) and the inputs of these delta-modulator circuits could be provided by existing bio-mimetic micro-electro-mechanical systems (MEMS) implementations of cricket filiform hairs (Krijnen et al., 2006). Furthermore, as shown in Supplementary Figure 8, the proposed model demonstrates robustness when subjected to substantial random permutations of its optimized parameter values. The model therefore may also be well-suited to a resistive memory array based implementation (Thomas, 2013) of its synaptic weight matrix where, due to inherent programming randomness (Ambrogio et al., 2014), weight values are subject to similar deviations from their desired values. Despite this deviation from the exact biology, two predictions from electrophysiological studies were found, in our model, to be of computational importance in the efficient detection of the simulated attacking predators. Specifically the presence of lateral inhibitory connections between the directionally selective interneurons predicted in Miller et al. (1991) as well as the global regulation, or background subtraction, mechanism proposed in Dupuy et al. (2012). These results raise the exciting question as to what else our neural network model might tell us about the mechanisms at play in the real cercal system. For example, could the lateral excitatory connections between directionally sensitive interneurons or the lateral connections that encourage the diversion in activation between air current speed encoding neurons also exist in the biological system?

While this cercal system escape response model was inspired by research pertaining to the cricket terminal abdominal ganglion, it shares a number of common principles found in other perception systems and across species. Most marked are the parallels between it and features of the *Drosophila* visual system that has recently come under intense investigation. For instance, just as the filiform hairs on cricket cerci sort local air-currents into one of four angles (45, 135, 225, or 315°); the elementary motion detection circuits in the lamina and medulla visual system layers of *Drosophila* sort local motion into one of four cardinal directions (up, down, left, and right) (Maisak et al., 2013). Also, just as specific regions of the TAG integrate spikes from the sensory neurons of the cerci into distinct regions corresponding to direction, the array of elementary motion detection circuits integrate their global activity in specific regions of the *Drosophila* lobular plate—forming a similar directional and spatial feature map of its visual scene to that in the cricket TAG. Furthermore, in a similar fashion as to how the seven interneurons in our TAG model form a compressed representation of the thousands of spikes triggered on the two cerci, *Drosophila* boils down the equally high dimensional space of its original optical input into a representation of twenty-seven interneurons (Wu et al., 2016). Each of these interneurons, as in our model too, encode certain properties of the visual scene. One of the most interesting parallels with the optimized TAG model is the observation that the giant fibre neuron of *Drosophila*, that descends to the animals motor ganglion to initiate a jumping escape response, sums excitatory and delayed inhibitory input from two of these interneurons; one encoding stimulus speed and the other stimulus size (Ache et al., 2019). This resonates with the excitation and delayed (inline with Steinmann and Casas, 2017) inhibition from the *fast* and *slow* neurons in our model. These findings point to the application of common architectural principles across sensory modalities and between different species. This observation mirrors similar findings whereby striking parallels have been drawn between the common computing principles and neural circuits applied in the visual systems of insects and mammals—in spite of the fact that they have been evolving independently for over half a billion years (Borst and Helmstaedter, 2015).

In this paper we have proposed a neural network model of the cricket cercal system escape response, based on decades of research into the neurobiology of this system. The model was optimized using the backpropagation algorithm and applied to the task of detecting the signature of a simulated attacking predator. When compared with the multi-layer perceptrons, indicative of a generic deep learning approach, it was found that the proposed TAG model was able to obtain the same performance with between one and two orders of magnitude fewer synaptic parameters. This advantage was maintained when comparing against pruned and sparsified models too. The bio-inspired model architecture was also deemed to be further orders of magnitude more efficient according to the Akaike information criterion that measures the trade-off between model performance and model complexity. Additionally, based on the inherent structure of the logical relations between the neurons of the model, a first-order structural interpretation was made of the models optimized synaptic parameters. These key results indicate the latent potential in incorporating bio-inspired architectures into neural network models for improving their memory efficiency and also their interpretability—two of the major drawbacks in the application of deep learning approaches based on the universal approximation theorem (Cybenko, 1989). Future work on this modeling project should focus on the extension of the model to second-order behaviors through the incorporation of temporal properties in the neuron activation functions and the investigation of techniques such as backpropagation-through-time (Werbos, 1990) and three-factor learning rules (Bellec et al., 2020) for the determination of the model parameters. Not only will this allow temporal sequences to be input to the model instead of static snapshots, but could provide further insights into the workings of the biological system and shed further light on other predictions regarding it—its potential use of coincidence detection mechanisms for example (Mulder-Rosi et al., 2010).

What is clear is that, contrary to the prevailing directions in deep learning, intelligence in animal nervous systems is more than learning. Rather, innate architectures, discovered over the course of evolution and regularized by material and energy constraints, provide built-in solutions for many tasks that share common characteristics to those being addressed via deep learning approaches. Further to looming detection as discussed here, more complex behaviors, courtship, and burrowing for example, are also understood to be hardwired from birth (Tinbergen, 1951; Weber and Hoekstra, 2009). With the advent of recent methods that permit increasingly detailed study of animal nervous systems, ranging from electron microscopy based connectome reconstruction (Takemura et al., 2017) to optogenetic-based electrophysiology (Deisseroth, 2011), subsequent years promise that the neural architectures present in biological nervous systems can be more readily discovered and then transferred into neural network models. An important question to address with future work into the development of such bio-inspired neural network architectures is how an approach like this can be scaled to larger models and more complex tasks. Could, for instance, many function-specific bio-inspired modules optimized through backpropagation, like that presented here, be inter-linked in a larger topology to arrive at something that resembles an “artificial nervous system” rather than an artificial neural network?

## Data Availability Statement

The raw data supporting the conclusions of this article will be made available by the authors, without undue reservation.

## Author Contributions

TD, JM, and JC developed ideas for the proposed model. TD implemented the neural network models in PyTorch as well as the statistical input model. All of the authors participated in the writing of the paper.

## Conflict of Interest

The authors declare that the research was conducted in the absence of any commercial or financial relationships that could be construed as a potential conflict of interest.
