# Supplementary material for: Bio-Inspired Architectures Substantially Reduce the Memory Requirements of Neural Network Models
Source: Front Neurosci. 2021 Feb 23;15:612359. doi: 10.3389/fnins.2021.612359 (PMC7940538; doi:10.3389/fnins.2021.612359)
Supplement: Supplementary file 1 [file Data_Sheet_1.PDF]

Bio-inspired architectures substantially reduce the memory requirements of neural network models - Supplementary material

January 12, 2021

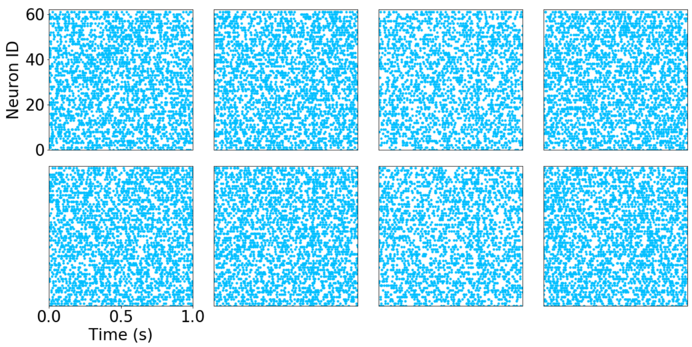

(a)

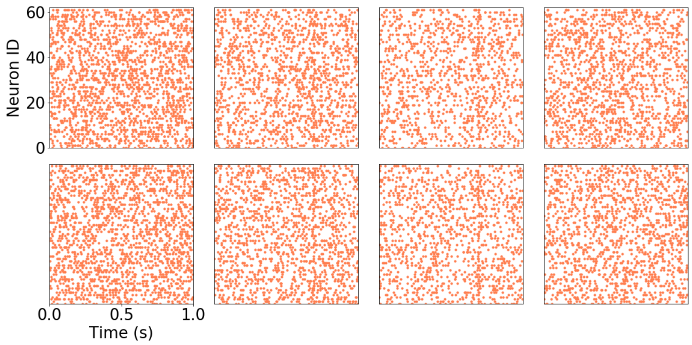

(b)

Figure 1: The raster plots of all of the sensory neurons, corresponding to those nested below the hairs, due an 'easy' to detect simulated attack with low background air intensity and a large fast attacker. From left to right the columns show the activity of the hairs oriented to 45°, 135°, 225°and 315°. The top row correspond to the hairs on the left cerci and the bottom row to the hairs on the right cerci. Note the vertical stripes of points at 700ms into the simulation in the central four panels which correspond to the attack. (a) As blue points, the spike-times of all of the slow (low frequency) hair sensory neurons are plotted. (b) As orange points, the spike-times of all of the fast (high frequency) hair sensory neurons are plotted.

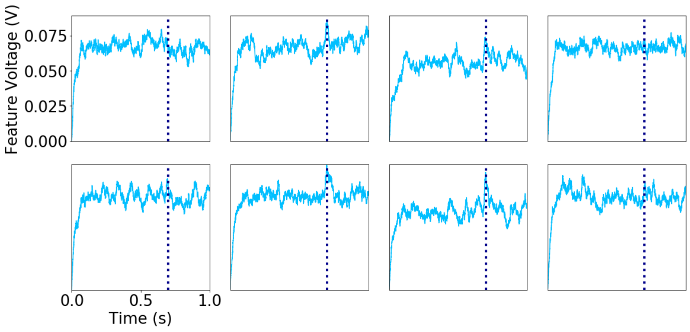

(a)

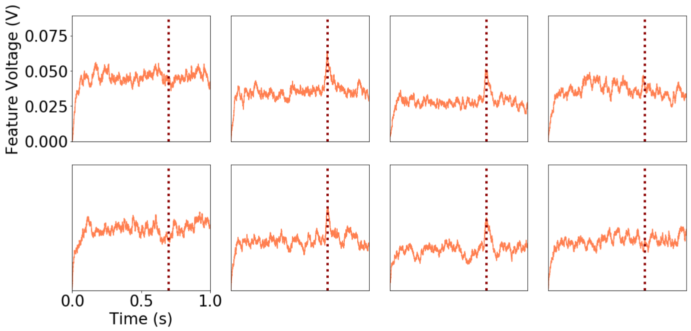

(b)

Figure 2: The membrane voltage plots of all of the sensory neurons, corresponding to those nested below the hairs, due the same 'easy' to detect simulated attack, with low background air intensity and a large fast attacker, that generated the raster plot in Appendix 1a. From left to right the columns show output voltages of the input layer neurons being excited by the hair populations oriented to 45°, 135°, 225°and 315°. The top row correspond to the input layer neurons receiving input from the hair populations on the left cerci and the bottom row to the populations on the right cerci. The vertical line drawn at 700ms denotes the attack time. The output voltages at this time, of all sixteen neurons, are used as the features that describe each data point in the training and testing of the TAG model in main paper. (a) As a continuous blue signal, the output voltages, denoted as feature voltage in the plot to denote the fact they are used as input features to the TAG model, of all of the slow (low frequency) input neurons are plotted. (b) As a continuous orange signal, the output voltages, denoted as feature voltage in the plot to denote the fact they are used as input features to the TAG model, of all of the fast (high frequency) input neurons are plotted.

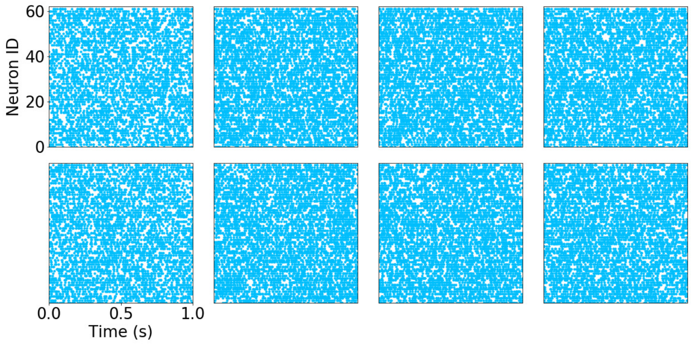

(a)

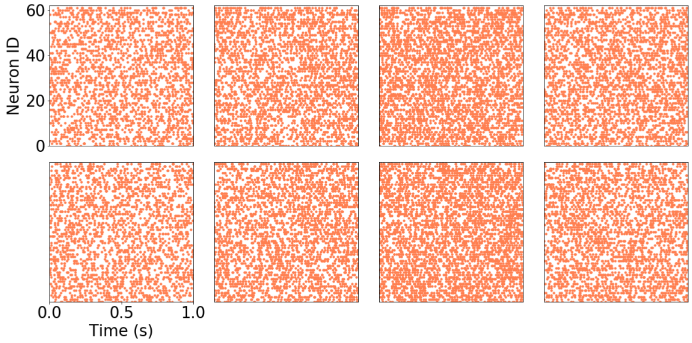

(b)

Figure 3: The raster plots of all of the sensory neurons, corresponding to those nested below the hairs, due a 'hard' to detect simulated attack with high background air intensity and a smaller slow attacker. From left to right the columns show the activity of the hairs oriented to 45°, 135°, 225°and 315°. The top row correspond to the hairs on the left cerci and the bottom row to the hairs on the right cerci. (a) As blue points, the spike-times of all of the slow (low frequency) hair sensory neurons are plotted. (b) As orange points, the spike-times of all of the fast (high frequency) hair sensory neurons are plotted.

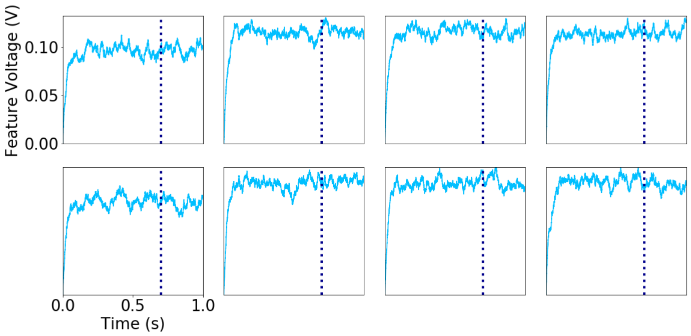

(a)

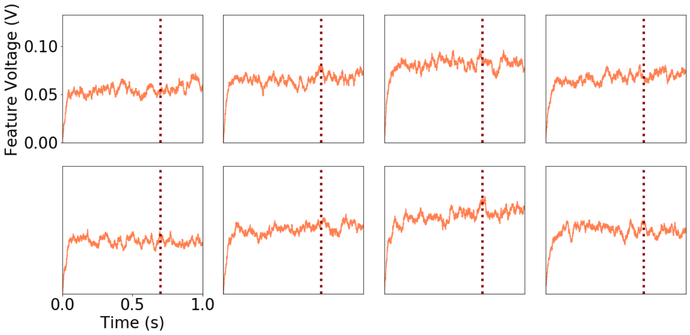

(b)

Figure 4: The membrane voltage plots of all of the sensory neurons, corresponding to those nested below the hairs, due the same 'hard' to detect simulated attack, with low background air intensity and a large fast attacker, that generated the raster plot in Appendix 3b. From left to right the columns show output voltages of the input layer neurons being excited by the hair populations oriented to 45°, 135°, 225°and 315°. The top row correspond to the input layer neurons receiving input from the hair populations on the left cerci and the bottom row to the populations on the right cerci. The vertical line drawn at 700ms denotes the attack time. The output voltages at this time, of all sixteen neurons, are used as the features that describe each data point in the training and testing of the TAG model in the main paper. (a) As a continuous blue signal, the output voltages, denoted as feature voltage in the plot to denote the fact they are used as input features to the TAG model, of all of the slow (low frequency) input neurons are plotted. (b) As a continuous orange signal, the output voltages, denoted as feature voltage in the plot to denote the fact they are used as input features to the TAG model, of all of the fast (high frequency) input neurons are plotted.

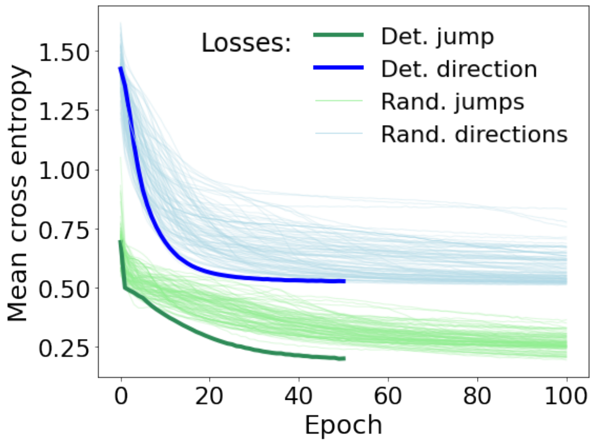

Figure 5: **Learning curve of the TAG model using the deterministic (single thick lines) initialisation and random (thin lines) initialisation**. The deterministic initialisation uses initial parameter values equal to either 1, 0 or -1 that correspond to the logic of the bio-inspired architecture whereas the random approach samples the initial values from a normal distribution centred on zero and with a standard deviation of one. Green and blue colours indicate the loss due to the mean binary cross-entropy of the jump neuron and the cross-entropy of the directional neurons over each training epoch. Greater variability and longer convergence is seen in the case of random initialisation.

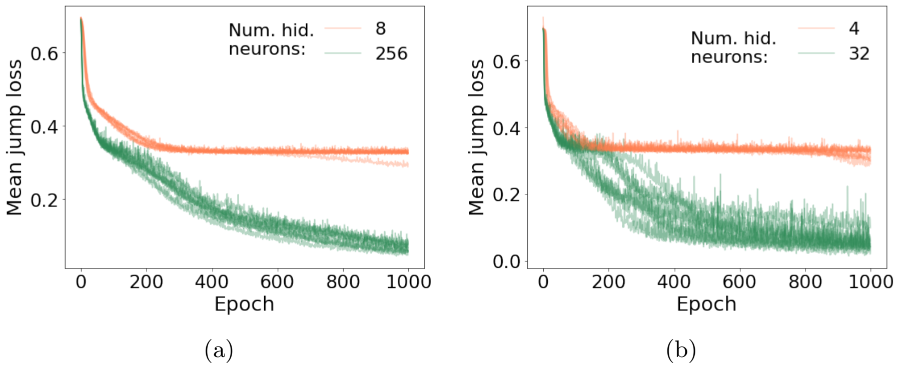

Figure 6: **Superimposed learning curves (ten iterations) of the comparative multi-layer perceptrons for small (orange) and larger (green) versions of the single and hidden layer models** (a) For the case of a single hidden layer MLP with 8 and 256 neurons in the hidden layer. (b) For the case of a three hidden layer MLP with 4 and 32 neurons in each hidden layer.

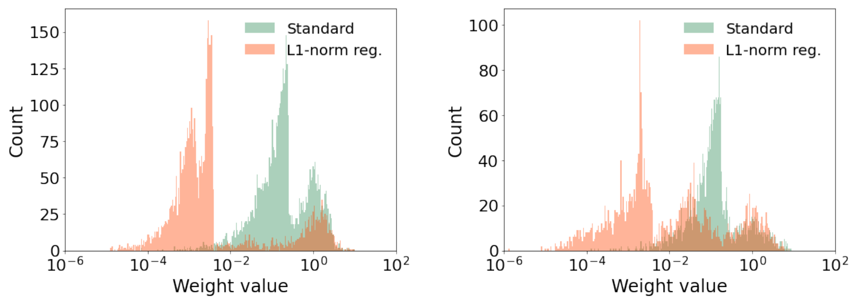

(a)

(b)

Figure 7: **Histograms of the standard (green) and L_1-norm regularised (orange) MLPs absolute weights after training, using 250 logarithmically spaced bins over a range of four decades**. (a) For the case of a single hidden layer MLP with 256 neurons in the hidden layer. (b) For the case of a three hidden layer MLP with 32 neurons per hidden layer.

Supplementary Note 1 - Model functional interpretation

Although it arises as a direct consequence of the structural properties of the model, another interpretation of the model can be made from a functional perspective - therein how the neurons activate when presented with input data-points. In order to build up this picture, the probability distributions of interneuron activations over the 1000 test data-points are plotted and discussed.

The activation distributions of the directionally selective interneurons are shown in Fig. 9. Resulting from the excitatory input from the relevant feature map neurons, and the response shaping due to the lateral connections, each of these distribution shows a clear bi-modal response. Specifically, for a *background direction* in the preferred direction of the interneuron, the activation is strongly positive, and, for a *background direction* coming from any other angle, the response is strongly negative. Functionally these interneurons are seen to implement a 'one-hot' encoding of the *background direction*, reminiscent of winner-take-all computational mechanisms that are equally achieved through competitive lateral interactions between neurons [1, 2].

The fascinating computation carried out by the *slow* and *fast* interneuron pair is depicted in Fig. 10. Specifically the interneurons are drawn in their 'unrolled' format and the intermediate and final activation distributions of the *slow* and *fast* neurons are plotted as insets. The intermediate activation distribution of *slow* spans predominantly positive values, reflecting its strong excitatory input from the feature map neurons in the input layer and the comparatively weaker input from the *glob* neuron. In this intermediate stage therefore, its

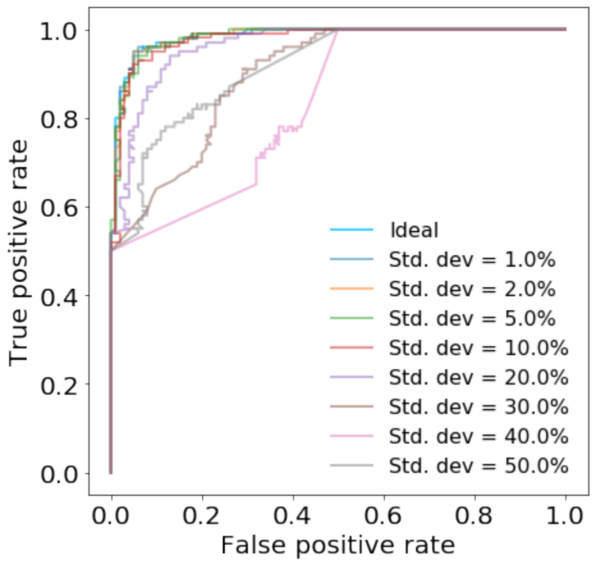

Figure 8: **The effect of random perturbations to the parameter values on the receiver operation characteristic curves.** After training a TAG model, random perturbations were introduced by re-sampling each parameter value from a normal distribution centred on its previous value using a range of standard deviations. The average ROC curve over ten iterations is plotted. The ROC curve is seen to exhibit minimal degradation up to a standard deviation of 10%. This high level of robustness when faced with considerable perturbations to its parameter values is likely owed to the fact that model structure is the dominant factor in determining its outputs as opposed to precision in its parameters.

activation is proportional to the slow air current intensity coming from behind, given a low background intensity. Since *glob* is inhibited by air current stimuli coming from behind the animal, *slow* is seen to, on average, activate most due to input data points that are due to an attack - although the distribution of responses due to ambient conditions has a considerable overlap. The intermediate attack and ambient activation distributions for the *fast* interneuron also exhibit a significant overlap. However, due to the particular sensitivity of this neuron to fast air currents coming from 135° and 225° from respective left and right cerci, one of the hallmarks of an attack modelled by the equations of the statistical model presented in the main paper, *fast* responds more positively to attack stimuli and more negatively to ambient stimuli on average. Through repulsive lateral interaction of these intermediate activations, the final activation distributions of *slow* and *fast* due to an attack are seen to respond more negatively and more positively respectively - reflecting the divergence between their post-synaptic connections to *jump* as shown in the connectivity tables in

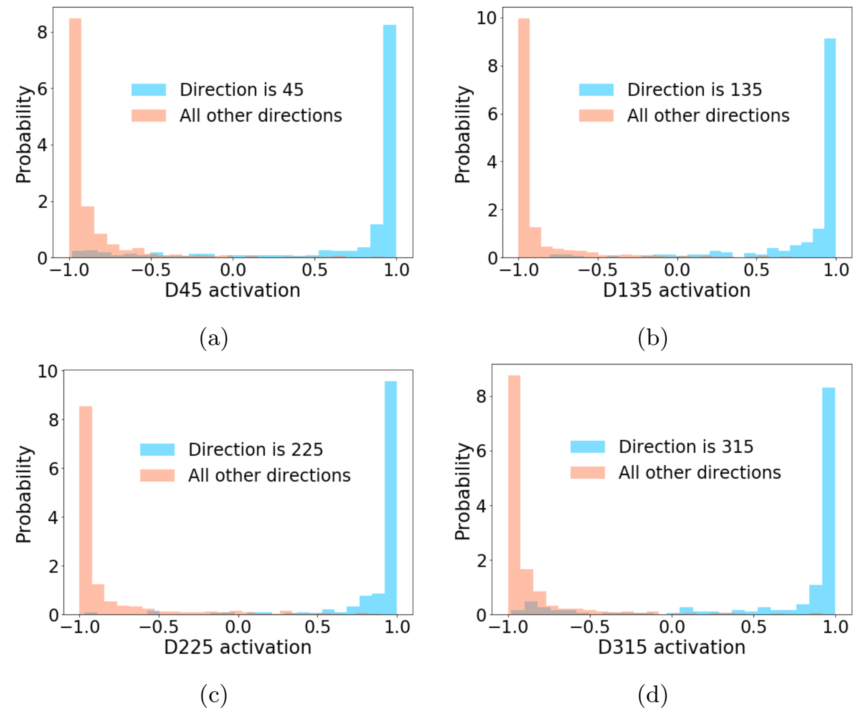

Figure 9: **The activation distributions over the** 1000 **test-points for each of the directional encoding interneurons**. The blue distributions show the hyperbolic tangent activations when the prevailing air-current direction was equivalent to the preferred direction of the sensory neurons that feed into the feature map neurons that each neuron receives excitation from. The red distributions correspond to when the prevailing direction of the air-currents was not in the neurons preferred direction. (a) Directional encoding neuron for air-currents from 45° . (b) Directional encoding neuron for air-currents from 135° . (c) Directional encoding neuron for air-currents from 225° . (d) Directional encoding neuron for air-currents from 315° .

the main paper.

Finally, to complete the functional picture, the activation distributions of *glob* under high and low background air current intensities and of *jump* when the model is presented with *attack* and *ambient* data points is shown in Fig. 11. Consistent with its proposed dual role as an inhibitor and as an excitor the *glob* activation responds with broad, largely negative or positive, activation distributions for low and high background air current intensities respectively. As reflected by the large area under the ROC curve of the optimised TAG model in the main paper, the eventual attack and ambient *jump* distributions over all of the test data-points for the sigmoidal *jump* neuron are well separated. The response is predominantly a binary one, with the output layer activating

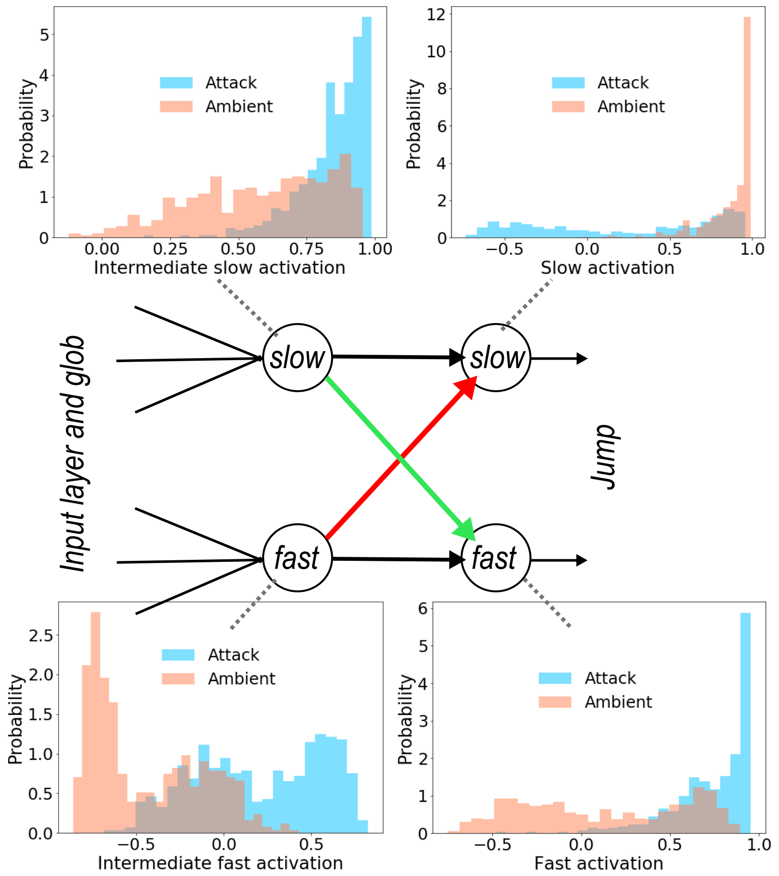

Figure 10: **The activation distributions over the 1000 test-points for each of the air current speed encoding interneurons in their intermediate and final, unrolled, states** The blue distributions show the hyperbolic tangent activations when the data-point pertains to an attacking predator and the red distribution to ambient conditions. (top left) The intermediate hyperbolic tangent activation of the *slow* neuron due to the feed-forward excitation from the feature map neurons. (top right) The final, unrolled hyperbolic tangent activation of the *slow* interneurons after the lateral inhibition from the intermediate state of the *fast* neuron. (bottom left) The intermediate hyperbolic tangent activation of the *fast* neuron due to the feed-forward excitation from the feature map neurons. (bottom right) The final, unrolled hyperbolic tangent activation of the *fast* interneurons after the lateral excitation from the intermediate state of the *slow* neuron.

very close to zero or one in most cases. Adaptation of the probability threshold, which is effectively a vertical line drawn through the distribution of Fig. 11b,

allows the tolerated false positive rate to be tuned.

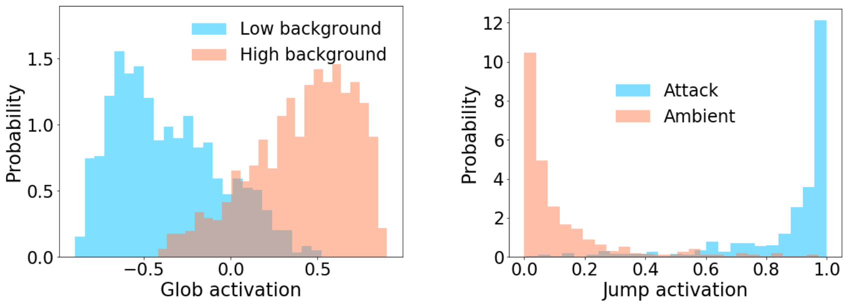

(a)

(b)

Figure 11: (a) The hyperbolic tangent activation of the *glob* neuron due to the feed-forward connections from the feature map neurons. The blue distribution shows the hyperbolic tangent activations for the test-points where the background air-current intensity was low and the red distribution when the background air-current intensity was high. (b) The sigmoidal activation distributions of the *jump* neuron for the (blue) attack test-points and (red) ambient test-points. A decision on whether to initiate a jump escape routine can be made by comparing the activation with a probability threshold along the x-axis.

References

[1] John Lazzaro, Sylvie Ryckebusch, Misha Anne Mahowald, and Caver A Mead. Winner-take-all networks of o (n) complexity. In *Advances in neural information processing systems*, pages 703–711, 1989.

[2] Wolfgang Maass. On the computational power of winner-take-all. *Neural computation*, 12(11):2519–2535, 2000.
